# A weather-forecast driven early warning system for wheat blast disease: User-centered design, validation, and scaling in Bangladesh and Brazil

**DOI:** 10.1016/j.cliser.2025.100589

**Published:** 2025-08

**Authors:** Timothy J. Krupnik, José Mauricio Cunha Fernandes, Felipe Vargas, Emerson Medeiros Del Ponte, Khaled Hossain, Mustafa Kamal, Mutasim Billah, Md. Harun-Or-Rashid, Sk. Ghulam Hussain, Pawan Kumar Singh, Krishna Kanta Roy, Carlos Augusto Pizolotto, Md. Shah Kamal Khan, Willingthon Pavan, Golam Faruq

**Affiliations:** a International Maize and Wheat Improvement Center (CIMMYT), Dhaka, Bangladesh; b Empresa Brasileira de Pesquisa Agropecuária (Embrapa), Trigo, Brazil; c SensorOn, Estrada do Trigo, Brazil; d Departamento de Fitopatologia, Universidade Federal de Viçosa, Brazil; e CIMMYT, El Batan, Mexico; f Bangladesh Wheat and Maize Research Institute (BWMRI), Dinajpur, Bangladesh; g Cooperativa Central Gaúcha Ltda. (CCGL), Cruz Alta, Brazil; h Department of Agricultural Extension (DAE), Dhaka, Bangladesh; i Department of Agricultural and Biological Engineering, University of Florida (UF), Gainesville, FL, USA

**Keywords:** Early warning system, Decision support tool, *Magnaporthe oryzae* pathotype *Triticum*, Software design, Human centered design, Integrated pest management

## Abstract

We describe the user-centered design (UCD) of a numerical weather-forecast-driven early warning system (EWS) as a climate service for managing wheat blast, a fungal disease capable of causing complete crop yield losses that is strongly dependent on weather conditions. Our mixed-methods process was guided by stakeholder input on the design, testing, and refinement of the EWS from agricultural extension organizations, meteorological departments, and farmers’ groups in Bangladesh and Brazil, where concerns about blast disease risks are high. The UCD process led to a wheat blast disease prediction model, server systems, and user-facing enhancements, including an open-source dashboard (https://beattheblastews.net/) that displays historical, real-time, and forecasted weather data, along with geographically explicit disease predictions, to support informed decision-making on wheat blast management. We describe the back- and front-end design of the dashboard, which supports disease risk forecasting, hindcasting, and the dissemination of early warning advisories co-designed with user organizations. We validated the EWS through comparisons with field observations in both countries. Model results generally agreed with disease incidence records, and model hindcasting confirmed alignment with disease outbreak patterns in Bangladesh and Brazil. Collaboration between agricultural research, meteorological and extension organizations in developing and supplying weather forecasts, disease management advisories, and early warning systems—along with presenting hindcast validation results to stakeholders—led to the formal endorsement of the EWS in both countries. This process also enabled the registration and training of over 14,500 extension officers, lead farmers, and farmers' cooperative members who now receive advisories via email, SMS, agro-meteorological bulletins, smartphone applications, WhatsApp and social media messages. These tools support them in interpreting and sharing wheat blast early warnings with farmers to improve disease preparadness and management actions in both countries.

## Highlights

• Wheat blast is a serious fungal disease strongly influenced by weather conditions.
• User-centered design (UCD) of a weather-driven disease early warning system (EWS).
• Extension, meteorological agency, and farmers’ groups supported design and validation.
• The EWS was evaluated in Bangladesh and Brazil, where blast is a serious concern.
• The UCD enabled endorsement; the EWS has >14,500 registered users in both countries.

**Practical Implications**

Wheat blast disease is a major concern in South America and South Asia, where prevailing weather conditions drive infection rates and can cause sudden and severe crop losses (Montes et al., 2022, Pequeno et al., 2024). This study outlines a user-centered design (UCD) approach employed to develop and validate a weather forecast-driven Early Warning System (EWS) for wheat blast in two countries affected by unexpected outbreaks. In Brazil, wheat blast has been a longstanding threat, with periodic outbreaks resulting in substantial production losses (Fernandes et al., 2017). In Bangladesh, the disease emerged unexpectedly in 2016, marking its first occurrence outside South America, affecting over 15,000 ha in under a month and raising food security concerns across South Asia (Malaker et al., 2016, Montes et al., 2022). Over the past nine years, meteorological departments, agricultural research institutes, extension orgaizations, and farmers' organizations in Bangladesh and Brazil have collaborated to develop and disseminate weather-based disease management advisories for wheat producers. While this study focuses on these two countries, the EWS development framework described in this paper offers a customizable model for agricultural climate services in other regions.

The UCD approach in Bangladesh and Brazil included stakeholder scoping, data collection and analysis, and system co-design, validation, and delivery stages. This led to a meteorological forecast-driven EWS that provides farmers with information to improve decision-making and implement timely preventative measures against disease development and outbreaks. During the scoping stage, CIMMYT identified and convened stakeholders in both countries to establish shared goals for the EWS. In Brazil, the EWS was developed through collaboration between CIMMYT, *Empresa Brasileira de Pesquisa Agropecuária* (EMBRAPA, Brazil’s largest agricultural research organization linked to the Ministry of Agriculture) and the *Central Cooperativa Gaúcha Ltda*. (CCGL), one of Brazil's largest farmer cooperatives. In Bangladesh, CIMMYT worked with the Department of Agricultural Extension (DAE), the Bangladesh Meteorological Department (BMD), the Bangladesh Agricultural Research Institute (BARI) and the Bangladesh Wheat and Maize Research Institute (BWMRI). The next UCD stage involved collecting qualitative data to assess the needs of EWS users at DAE and CCGL. This was followed by an EWS system design phase that included developing weather forecast-driven disease models and data products, and integrating stakeholder feedback to create an interactive disease risk visualization dashboard and advisory system (https://beattheblastews.net/).

Validation with national agricultural research institutes led to refinements in the system that enhanced its components before and after stakeholder endorsement. Validation processes also supported organizational collaborations that resulted in user registration, training, and usability testing during the delivery stage. As wheat blast is influenced by weather conditions, the EWS uses real-time, historical, and forecasted weather data to predict outbreaks based on environmental conditions favorable to the disease. The system's back-end includes an application programming interface (API) with a gridded weather forecast-driven disease risk model that triggers location-specific advisories when and where disease risks are detected. Model evaluation compared predicted disease incidence with historical field observations in both countries with satisfactory results.

In Bangladesh and Brazil, dissemination efforts ensured farmers received wheat blast management advisories through extension services. In Southern Brazil, where CCGL is active, approximately 5,000 farmers and 1,000 field agronomists registered in the EWS receive SMS alerts and advisories. Advisory details are also accessible via CCGL’s WhatsApp groups and SmartCoop web application (https://app.smart.coop.br/), which has approximately 15,000 subscribers. In Bangladesh, over six years, 8,576 extension officers and farmers' group leaders were registered in the EWS and completed trainings on how to interpret and share advisories. Advisories are distributed through email, SMS, and weekly agro-meteorological bulletins published on DAE’s online portal, with the wheat blast disease prediction dashboard also available through BMD’s and DAE’s websites (https://live8.bmd.gov.bd/ and https://www.bamis.gov.bd/).

For researchers developing agricultural climate services, particularly for disease management, the design process outlined in this paper can be adapted to suit different regions, countries, and agricultural pests. The EWS’s capacity to deliver early warnings using weather forecasts enables proactive rather than reactive disease management. Extension services can use the EWS to enhance advisories for farmers in both countries. The system could also be expanded through use of its API to include other countries where wheat blast poses an established or emerging risk.

Perspectives on the practical applications of this work in Bangladesh include comments provided by agricultural extension officers and farmers, respectively:

*“The training we received on using the EWS has greatly enhanced our ability to support farmers. The system's forecasts are reliable, and the advisory dissemination methods are effective in reaching even the most remote areas. After getting the EWS advisory forecasting wheat blast disease, we informed farmers to use disease management strategies. When we received a message indicating no risk, we informed them not to take any action. This helped farmers decide when to spray and when not to, benefiting them economically and promoting safe food production.*” An anonymous extension agent from DAE in Bangladesh.

*“Receiving timely alerts through the EWS has been a game-changer. We can now take preventative measures before the disease spreads widely. After getting messages from extension officers, we took initiative to control the disease. When there is no disease, the forecast helps us save money because we do not need to apply pesticides. It's like having a safety net for our crops.”* An anonymous wheat farmer from blast disease-prone area in southern Bangladesh.

Based on this work, key insights for the UCD of weather-driven EWSs for crop diseases include:

• **Frameworks can guide, though customization is necessary:** The design framework outlined in this paper provides a valuable guideline for research and stakeholder engagement in developing climate- and weather-driven EWSs for other crop diseases. Effective warning systems require customization, refinement, and country-specific adaptations, which are integral to UCD processes. In Bangladesh and Brazil, stakeholder types and preferences differed, prompting tailored dissemination methods and unique advisory content to address the specific needs expresssed in each country, and among extension organizations and farmers’ groups.
• **Stakeholders are key to model development:** Active stakeholder involvement throughout the UCD process was essential to ensuring the EWS addressed extension users’ practical needs. Key informant interactions, focus groups, validation processes convened by a national wheat blast task force, as well as interactive training sessions, enabled effective communication and feedback between developers and users. This engagement fostered organizational cooperation, improving the system’s functionality.
• **Data and evidence are vital for EWS endorsement by stakeholders:** Comparing EWS-derived outbreak notifications with historical climate data and wheat blast observations from BARI, BWMRI, and EMBRAPA demonstrated reliable model performance. These results validated the model’s accuracy in predicting disease occurrence and were instrumental in stakeholder validation workshops that were integral to the UCD process and securing formal EWS endorsement in Bangladesh and Brazil.

## Introduction

Wheat, grown on over 221 million hectares globally (FAOSTAT, 2023), provides 20 % of daily protein and calories consumed (Shewry and Hey, 2015), but is highly susceptible to diseases, particularly in tropical and subtropical regions where climatic conditions favor fungal epidemics that can cause major losses for farmers (Pequeno et al., 2024, dos Santos et al., 2022). Wheat blast, caused by the Triticum lineage of the fungus *Magnaporthe oryzae* (MoT) (Urashima et al., 2009, Montes et al., 2022, Mottaleb et al., 2018, Mottaleb et al., 2023, Pequeno et al., 2024), affects wheat and other grasses, and is among the most challenging diseases to manage (Cruz and Valent, 2017, Ascari et al., 2021). Disease severity depends on climatic conditions, cultivar susceptibility, and the timing and location of infections at the landscape level, within crop fields, and on plant organs (Goulart et al., 2007). High base temperatures, precipitation, and relative humidity accelerate spore development and infection (Calvero et al., 1996, Fernandes et al., 2017, Pequeno et al., 2024). Ideal temperatures for infection range from 25–30 °C but can initiate at 12–15 °C under prolonged wetness (Cardoso et al., 2008, Fernandes et al., 2017, Montes et al., 2022).

Wheat blast, capable of causing complete yield losses, was first identified in Brazil in 1985 and later spread to Bolivia, Paraguay, and Argentina, where conditions support periodic outbreaks (Fernandes et al., 2017, Fernandes et al., 2021). In 2016, wheat blast was identified outside the Americas for the first time, infecting over 15,000 ha in Bangladesh and reducing production by 8,205 tons. This costfarmers USD 2.1 million in crop losses (Malaker et al., 2016, Mottaleb et al., 2018). Concerns arose over the potential spread of blast to India, Nepal, and Pakistan, where it could threaten food security for 300 million people dependent on 100 million tons of wheat annually (Duveiller et al., 2016, Chowdhury et al., 2017). Recently, wheat blast was also identified in Zambia, raising fears of outbreaks in Africa (Tembo et al., 2020, Singh et al., 2021).

Despite advances in wheat blast-resistant cultivars, even the most resistant varieties cannot fully control the disease under field conditions (Cruz et al., 2016, Singh et al., 2021, Krupnik et al., 2024). Fungicides, which are typically recommended as a secondary defense, are most effective when applied early, before symptoms appear to the naked eye. Converns however remain about the profitability of fungicide use in smallholder farming contexts, in addition to their environmental safety, particularly for post-infection management under favorable conditions (Cruz et al., 2019, Ascari et al., 2021). In Bangladesh, agricultural extension services typically advise avoiding susceptible cultivars, sowing early to reduce crop exposure to late-season weather risks, and using healthy seeds, though these strategies alone are unlikely to be sufficient for comprehensive control.

In response, early warning systems (EWSs) for disease management are an emerging topic in agricultural climate services, particularly for smallholder farmers and with respect to disease management (Bharwani et al., 2024, Charalampopoulos and Droulia, 2024, Mason et al., 2022, Matengu et al., 2023, Salam et al., 2019, Smith et al., 2024). Use of climate and weather information to predict and advise farmers on wheat blast management is a critical area of study (Calvero et al., 1996, Fernandes et al., 2021, Pequeno et al., 2024). However, research on user-centered design (UCD) approaches to EWSs, corresponding software and advisory structure development, and their application in disease management within agricultural climate services remains limited (Charalampopoulos and Droulia, 2024). This constrains capacity to address challenges such as wheat blast, which is particularly difficult to detect in its early stages.

Because blast infections are nearly invisible to the naked eye (Cruz and Valent, 2017), farmers often detect wheat blast too late to prevent widespread damage. To address this problem, disease prediction models have been developed to forecast outbreak risks (Fernandes et al., 2017, Fernandes et al., 2021, Montes et al., 2022, Pequeno et al., 2024). However, these models are rarely evaluated using observed rather than modeled data and have not been integrated into operational, software-based, automated early warning systems capable of supporting disease management advisory dissemination—a gap this study addresses.

Using a UCD process, we explain how this approach facilitated collaboration among agricultural extension services, meteorological agencies, and farmers’ groups in developing software for a numerical weather-model-driven wheat blast EWS, dashboard, and advisory dissemination system in Bangladesh and Brazil, the epicenters of blast in South Asia and South America, respectively. Responding to national policies requiring the validation of early warnings and agricultural decision support tools, we also describe how model predictions were compared to field observations of disease incidence in both countries using a hindcasting approach using historical weather data. This flexible approach to UCD provides framework for the development of similar climate services in agriculture that can be applied in other regions and country contexts.

## Methods

### Study areas

#### Bangladesh

While rice is Bangladesh’s primary staple, wheat consumption is expected to rise by 6 % to 8.4 million tons in the coming years due to growing demand from restaurants, households and bakeries (USDA, 2022). Summer rainfall is high and winter rainfall is low in Bangladesh. Winters are marked by high humidity conditions, with average minimum temperatures from 11 to 16 °C (Wheat Atlas, 2022). Wheat is sown after monsoon season rice is harvested from late autumn forward, typically on well-drained soils, between November and December (Krupnik et al., 2015a, Krupnik et al., 2024). Wheat’s most challenging production environments are found in the south of Bangladesh near where the first incidence of wheat blast was recorded (Malaker et al., 2016), where both high temperatures and disease risks constrain yield (Krupnik et al., 2015b, Schulthess et al., 2019).

#### Brazil

Wheat production in Brazil is rising (FAOSTAT, 2023), partly due to government-backed efforts to boost self-sufficiency and exports. Annual consumption is approximately 12.8 million tons, with half imported (Torres et al., 2022). Wheat is grown in three main regions: the temperate south, the subtropical central west, and the tropical central Cerrados at above 800 m in altitude. The latter has a tropical Savannah climate with dry and rainy seasons. The dry season runs from May to September, averaging 30 °C with minimal rainfall; while the rainy season spans October to April, with cooler temperatures and more rainfall. Rainfed wheat is typically sown from late February to mid-March. Wheat blast is endemic to this region, resulting in periodic and often significant outbreaks (Torres et al., 2022).

### User-centered co-design process

**Fig. 1:**
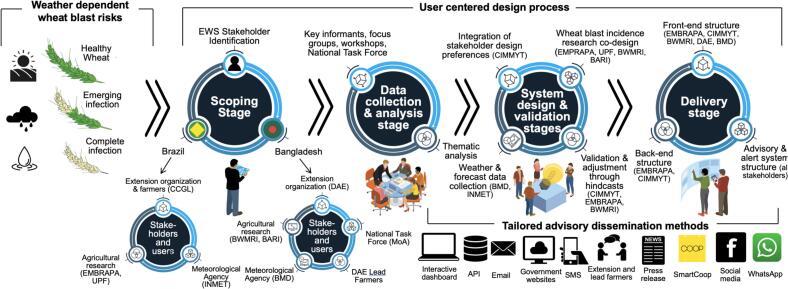
Weather dependent wheat blast risks and the user centered design software design process utilized in Bangladesh and Brazil to develop the numerical-forecast driven early warning system.

We used a UCD approach to develop an EWS that integrates the interests of agricultural extension services, meteorological agencies, and farmers’ groups with validation modeling and practical application as an agricultural climate service (Fig. 1). Wallach and Scholz (2012) outline five generalized UCD stages software for development, which we applied to stakeholder co-design of the weather-forecast-driven wheat blast EWS described below and in our results.

(1) **Scoping***:* This stage took place in 2016–2017. We identified and convened relevant stakeholders in agricultural research and extension agencies, meteorological services, and farmers’ groups in Brazil and Bangladesh, and engaged them in discussions to define EWS goals.
(2) **Data collection and analysis***:* During this stage from 2017 to 2019, key informant interviews, focus groups and workshops, including meetings of the National Wheat

Blast Task Force in Bangladesh, were used to collect unstructured qualitative data from the stakeholders described in Section 3.1. In all interactions, detailed notes were taken. Thematic analysis (Braun and Clarke, 2006) was used to interpret qualitative data, identify stakeholder attributes as EWS users, and guide the practical design of the EWS and advisory system.

(3) **System Design***:* This stage took place between 2019–2020 and encompassed both back-end and front-end EWS software design. The back-end focused on collecting weather and disease incidence data from collaborating stakeholder organizations to generate datasets for model validation and integration of the disease model, observed data, and weather forecast outputs on the server side. The front-end addressed stakeholder requests by developing a user-responsive interactive dashboard and an automated, location-specific advisory dissemination system supported by an API.
(4) **Validation***:* In practice, the design and validation stages are iterative and intertwined (Wallach and Scholz, 2012). To improve the software, the EWS’s back- and front-end components were validated through weather and disease hind-casting approaches that were conducted from 2019 to 2021, along with presentations of model performance and interactive dashboard design and testing to and with organizational stakeholders.
(5) **Delivery**: From 2022 to 2023, we involved agricultural research and extension agencies in designing maps to visualize wheat blast outbreak risks. Work during this period also involved the development of wheat blast advisory and management logic for farmers. Organizational end-users received training in EWS software operation, interpretation of disease forecasts and consequent management advisories, and dissemination of early warnings to farmers. Usability testing, typically part of validation in UCD, was integrated into extension staff training during the delivery stage, iteratively informing improvements to back- and front-end software design.

### The wheat blast model

The wheat blast disease prediction model that drives the EWS comprises four components: (1) a sporulation favorability index, (2) a spore cloud density prediction model, (3) location-specific numerical weather forecasts, and (4) a disease alert distribution system.

#### The weather data-driven disease spore cloud density model

Infection risk is typically considered the primary limiting factor for epidemics when inoculum is abundant, with most bio-climatic disease models estimating infection efficiency based on plant organ wetness duration and air temperature (Magarey et al., 2005). Wheat blast outbreaks are mainly driven by inoculum accumulation and the dispersal of airborne spores (Fernandes et al., 2021). To model the impact of weather on wheat blast inoculum build-up, we applied a generic disease life cycle model (GDM) parameterized to simulate the disease (Fernandes et al., 2019). The GDM assumes the presence of alternative wheat blast hosts near wheat fields and a geographically uniform distribution of disease inoculum in the environment. The model’s climatic components are described in detail by Pequeno et al. (2024) and in the coefficients provided in Supplementary Materials 1. A short summary of major model components is provided below.

**Fig. 2:**
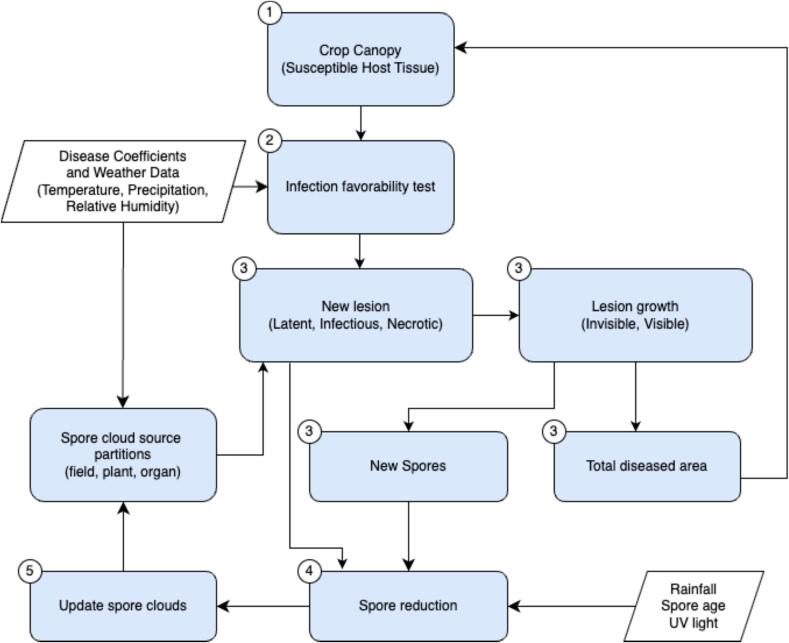
A schematic representation of the primary components of the generic disease model (GDM) described in this paper. Numbers in circles correspond to relevant summary Equations presented in this paper (with spore reduction including both Eqs. (4a), (4b), respectively).

The GDM is a mechanistic model that simulates wheat blast development over the growing season by calculating the daily formation of new spores in lesions across different wheat blast cohorts within a field. It uses hourly data on temperature, relative humidity, and precipitation as key drivers of disease progression (Fernandes et al., 2019). It is initialized using hourly weather data and disease coefficients (detailed in Supplementary Materials 1), which provide the environmental and biological parameters needed to model pathogen dynamics. The input files also define core simulation parameters, including the simulation start date, crop sowing date, and the initial value of the crop canopy object of the model, representing the crop’s susceptible host tissue available for infection (Fig. 2). This setup enables the model to track disease progression across a continuous host surface by combining environmental inputs with biological processes.

The GDM simulates wheat blast development by evaluating weather data to determine when conditions exceed critical thresholds for fungal growth and development including cardinal temperatures provided in the disease coefficients file. The temperature favorability function is located in the utility module and can be invoked by various model components as needed. During simulations, the model estimates total inoculum load by evaluating spore cloud development and spore density within a 1 m^3^ area above the crop canopy, represented by the spore cloud density variable (σ), where spores most likely persist and contribute to infection (Mousanejad et al., 2009). This is derived by aggregating contributions from three spore partitions: the crop organ-cloud (individual wheat organs), the plant-cloud (entire wheat plants), and the crop field-cloud (field-scale sources).

The resulting inoculum is deposited onto the crop canopy model object, representing the surface area available for infection. If environmental conditions meet infection thresholds, new lesions may form, as shown in Eq. (1),

(1) $$\text{New Lesions}=\left\{\begin{array}{cc} \sigma \cdot H\cdot \eta \cdot f_{T}\left(T_{mean}\right)\cdot f_{W}\left(D\right), & D\geq D_{th},\\ 0, & D<D_{th}. \end{array}\right)$$

where σ represents spore cloud density, H is the proportion of host tissue that is still healthy, and η represents wheat blast pathogen infection efficiency. The temperature favorability function of the model, $f_{T}\left(T_{mean}\right)$ is evaluated at the daily mean temperature, while the wetness favorability function, is $f_{W}\left(D\right)$, is evaluated as the duration of leaf wetness. Lastly, D represents daily wetness duration, and $D_{th}$ is the minimum wetness duration threshold for infection.

Following lesion initialization, the model simulates temporal development using logistic growth functions defined in the pest disease coefficients file (Supplementary Materials 1). These functions control the expansion of individual lesions and enable estimation of the cumulative affected host tissue, distinguishing between asymptomatic (invisible) and symptomatic (visible) stages of infection, as illustrated in Fig. 2. Each lesion progresses through distinct epidemiological stages: a latent period when the lesion is present but non-infectious; an infectious period during which spores are actively produced and released; and a necrotic period marked by tissue death and the end of spore production.

This stage-wise simulation provides a mechanistic representation of disease development, linking lesion-level physiological dynamics to broader epidemic patterns across the canopy. Spore production occurs only during the infectious phase and is simulated on the surface of each lesion cohort. Production is influenced by lesion age, temperature, relative humidity, and the maximum spore density per unit lesion area (cm^2^). The number of new spores produced, denoted as New Spores in Fig. 2, is calculated using a function that integrates these variables. This formulation captures the non-linear effects of environmental conditions and lesion physiology on spore production, and is shown in Eq. (2) which defines a logical test (sporeCondition) that must be met for spore production to occur,

(2) $$\text{sporeCondition}=\left(H>0.01\right)\;\wedge \;IP\;\wedge \;\left(W>W_{th}\right)$$

where H represents the proportion of healthy organ area, *IP* is a boolean indicating whether the day falls within the infection period, *W* denotes the daily wetness duration, and *W*_th_ is the wetness threshold.

The model analyzes weather data to determine when conditions exceed thresholds that influence wheat blast spore development. Spore production is driven by cohort age, the proportion of the cohort area affected by the disease, and air temperature. The model cycles through each cohort to estimate spore production per lesion, incorporating these variables (Pequeno et al., 2024). This process includes calculating the total number of lesions within each cohort, determining the spore production rate per lesion, and adjusting for cohort age using a trapezoidal function. It also accounts for the proportion of diseased tissue in plant organs as a crowding factor and adjusts outputs based on current temperature conditions. These factors are combined to calculate the total number of new spores produced for each cohort. Given the test condition calculated in Eq. (2), the daily number of new spores (newSpores) is shown in Eq. (3),

(3) $$\text{newSpores}=\left\{\begin{array}{cc} L\times s\times \tau \left(A\right)\times \kappa \left(H_{d}\right)\times f_{T}\left(T_{mean}\right), & \text{if}\;sporeCondition,\\ 0,\; & \text{otherwise}. \end{array}\right)$$

where *L* is the number of lesions in the cohort, *s* is the number of spores produced per lesion per day, and $\tau \left(A\right)$ is the value of a trapezoidal function based on disease cohort age measured in days since infection was triggered, computed through a trapezoidal function applied to the variable Age using the defined CohortAgeSet as input parameters. Lesions experience crowding effects at the plant organ, complete plant, and field levels; $\kappa \left(H_{d}\right)$ consequently represents the crowding effect on sporulation. Finally, $f_{T}\left(T_{mean}\right)$ represents the temperature favorability function called from the utility module.

The spores produced each day across all lesion cohorts are transferred to the model’s spore cloud compartment, which represents the pool of viable spores available for potential infection in a 1 m^3^ area above the canopy. These are aggregated-up to the level of the spatial grid required for the triggering of disease outbreak alerts (see Sections 2.3.2 and 2.4.3). Spore accumulation is updated daily based on total production, accounting for lesion development stages and losses due to rain wash-off, solar radiation, and age-related inoculum decline.

Light rainfall of less than 5 mm day^−1^ supports wheat blast infection, while heavier rainfall can dislodge conidia from lesions and wash away recently deposited spores from plant surfaces (Kim et al., 1990, Kim, 1994). Removal of spores from the spore cloud due to rainfall is therefore defined in Eq. (4a),

(4a) $$f\left(R\right)=\left\{\begin{array}{cc} \min\left(\frac{R}{80}\right),1 & \text{for}\;\;R\leq 15\\ 0 & \text{for 0}\leq R\leq 15 \end{array}\right)$$

where *R* is rainfall measured in mm day^−1^. As ultraviolet (UV) radiation can affect spore longevity in the atmosphere (Deacon, 2005), the model evaluates the effects of UV on the survival of airborne spores. UV sensitivity is modeled using daily temperature range (DTR) — the difference between maximum and minimum daily temperatures — as a proxy for cloud cover. High DTR values correspond to sunny conditions with strong UV exposure, leading to faster spore degradation. When DTR falls below a defined threshold (e.g., <9 °C), indicating cloudy conditions and reduced UV intensity, the model increases spore viability by one additional day to reflect lower UV-induced mortality (Li et al., 2018, Makowski et al., 2009, Pequeno et al., 2024, Lagomarsino Oneto et al., 2020). This is show in Eq. (4b),

(4b) $$\mathrm{If}\;\left(Tmax-Tmin\right)<\;9^{{}^{\circ}}C,\;thenSporeSurvivalDays=SporeSurvivalDays+1$$

where *T*max and *T*min represent daily maximum and minimum temperature, respectively, and *SporeSurvivalDays* is the baseline number of viable days for airborne spores. Conversely, spore removal from the cloud compartment is governed by an age-based decay process. As spores age, their viability declines, and the model progressively eliminates older spores to maintain a biologically realistic inoculum pool. This prevents overestimation of infection risk by discarding ineffective, aged spores. Age-based removal is implemented either through a FIFO buffer or via decay functions. Spores are only considered viable up to a maximum age, $A_{max}$, beyond which they are removed using Eq. (5):

(5) $$C_{\text{new}}=\sum_{a=0}^{A_{max}}C\left(a\right)$$

Here, $C_{\text{new}}$ is the calculated value for the number of total viable spores in a 1 m^3^ area over the crop canopy after age-based removal, $C\left(a\right)$ represents spores of exact the age a, and $A_{max}$ is the maximum viable age computed using the disease coefficients shown in Supplementary Materials 1. Simulation outputs are organized hierarchically across field, organ, and plant levels. Environmental variables — particularly rainfall, UV exposure, and spore age — jointly influence spore survival and infection potential.

To assess the risk of infection on wheat spikes, the model determines whether airborne spore concentrations exceed critical thresholds for infection. The model maintains an explicit spore cloud component and captures temporal variability in spore densities for secondary infection. This increases realism by linking lesion dynamics to broader disease development in polycyclic epidemics.

#### Observed and forecasted weather data integration

The EWS features a non-relational database storing weather data from an automated weather station network and five-day gridded forecasts from the Weather Research Forecasting (WRF) Model (Version 4.4.2, Boulder, USA) provided by the Bangladesh Meteorological Department (BMD, 17 km × 17 km) and the IBM Weather Company in Brazil (10 km × 10 km). It maps wheat-growing areas in both countries and uses weather data to simulate wheat blast spore cloud density and project future development. Gridded forecasts simulate spore density over crop canopies for the next five days, comparing results to a spore density threshold (Section 2.4.3). An API colors grid cells by risk level and sends notifications with management advisories to extension and farmers’ organizations in medium- or high-risk areas.

### Wheat blast model evaluation

In Bangladesh and Brazil, national agricultural policies require the integration of research findings with governmental extension services to validate decision support tools before public release (Planning Commission of Bangladesh, 2017, Schneider, 2014). We consequently involved relevant stakeholders identified during our Scoping UCD stage (Section 3.1) in data acquisition and model evaluation. In Bangladesh, this included BMD, the Bangladesh Wheat and Maize Research Institute (BWMRI), the Bangladesh Agricultural Research Institute (BARI), and the Department of Agricultural Extension (DAE) while *Empresa Brasileira de Pesquisa Agropecuária* (EMBRAPA), Brazil’s agricultural research and extension corporation linked to the Ministry of Agriculture and a range of farmers’ cooperatives, supported this process in Brazil.

#### Observed weather database

**Fig. 3:**
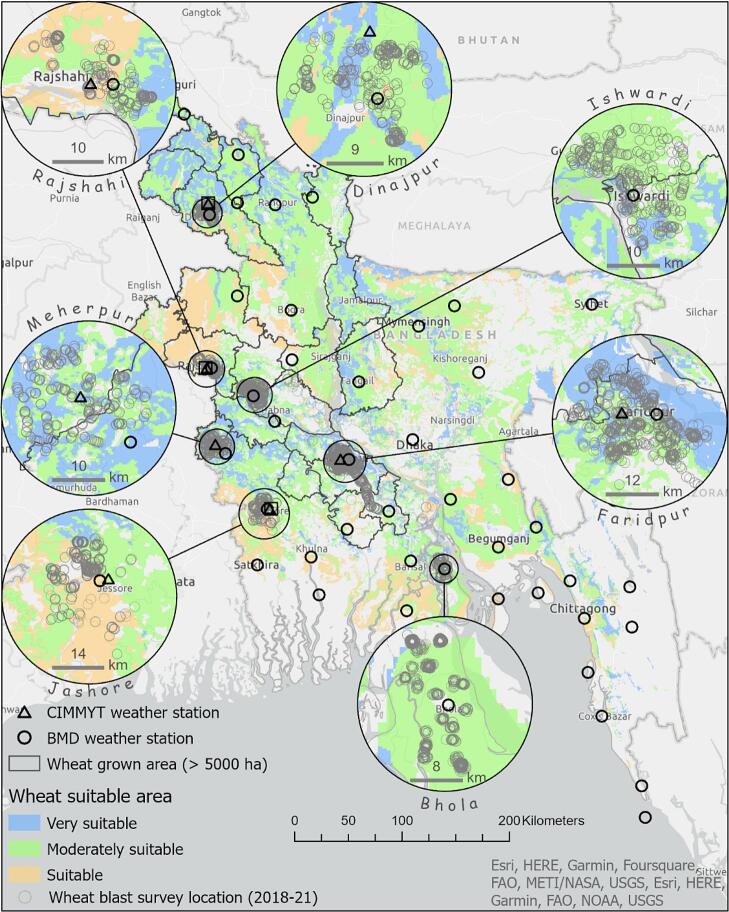
The study location including wheat-suitable growing areas in Bangladesh with the wheat blast survey locations and the automated meteorological stations.

In Bangladesh, BMD provided air temperature, relative humidity, and precipitation data from its synoptic station network (three-hour intervals). This was complemented by RX3000 weather stations (Hobo Onset, Bourne, MA, USA) installed by CIMMYT. These covered the area of interest at 15-minute intervals (Fig. 3).

**Fig. 4:**
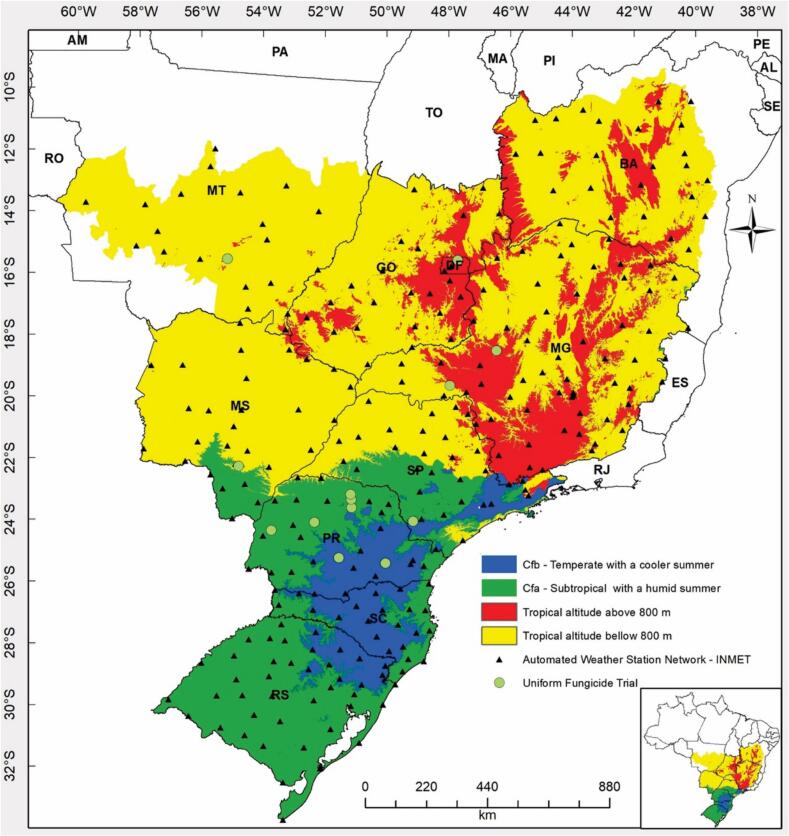
The study location in Brazil, showing agro-climatic classifications for rainfed wheat suitability zoning (colored areas). Triangles represent automated weather stations, and green circles indicate uniform fungicide trial locations.

In Brazil, EMBRAPA required the use of observed data from the *Instituto Nacional de Meteorologia*’s (INMET) automated weather station network (Moura and Fortes, 2016) for validations. Weather data were selected from stations within a 24.5 km radius of sampled wheat fields in Bangladesh and uniform fungicide trials in Brazil (Sections 2.3.2.1 and Fig. 3, Fig. 4).

Where only three-hourly observations were available (as with BMD stations), data were linearly interpolated to obtain hourly values. Observed weather data were stored in a non-relational database linked to a dashboard created to represent the data (Section 3.4.1).

#### Validation data

##### Observed wheat blast data

As part their annual disease surveillance efforts in Bangladesh, BWMRI trained and deployed enumerators to monitor up to 24 wheat fields during the advanced reproductive stages of wheat growth from March 2–11 in the 2018–2019, 2019–2020, and 2021–2022 wheat growing seasons. Observations took place within a 24.5 km radius of weather stations across seven major wheat-growing regions (Fig. 3). Fields were untreated with fungicides, lacked resistant varieties, and were spaced at least 200 m apart. This effort generated 304 observations of wheat blast incidence and severity, based on two transects and random sampling of at least 100 spikes per field in an ‘X’ pattern. Additional data on blast incidence and infection extent were obtained from Bangladesh’s DAE for each season from the 2015–2016 to 2021–2022 wheat growing seasons. In Brazil, EMBRAPA does not conduct systematic disease surveys as in Bangladesh. Instead, it provided data from its network of uniform fungicide trials (Fig. 4). The sampling procedure, described by Ascari et al. (2021), involved 14 blast-susceptible cultivars adapted to the region. We used only data from fungicide-free plots, where disease expression was most severe.

#### Wheat blast status and model validation

Disease incidence, defined as the proportion of infected spikes among all spikes evaluated, was assessed using 100 heads sampled from surveilled fields in Bangladesh and from uniform fungicide trials in Brazil. Incidence was classified as severe (>20 %) or non-severe (<20 %), aligning with critical-point yield loss models (dos Santos et al., 2022). A severe infection status is triggered when the model generated ≥5 notification alerts with incidence >20 % during the wheat heading stage.

To evaluate model performance, a confusion matrix was used, with rows representing predicted classes and columns representing actual classes. Results from Bangladesh and Brazil were categorized into four groups: (1) fields correctly classified as having wheat blast epidemic conditions (true positives), (2) fields correctly classified as not having epidemic conditions (true negatives), (3) fields without observed disease incidence but with model-generated alerts (false positives or type I error), and (4) fields with reported epidemics but no model alerts (false negatives or type II error). Metrics assessing model performance include:

• **Precision:** Indicative of model positive prediction accuracy, the proportion of true positive predictions among all positive predictions.
• **Negative Predictive Value:** The proportion of true negatives among all observed negative cases, reflecting the model’s reliability in identifying negative outcomes.
• **Accuracy:** The proportion of correctly classified true positives and true negatives among all predictions, representing overall model performance.
• **Sensitivity:** The proportion of true positives among all actual positive cases, measuring the model’s ability to identify positive disease incidences.
• **Specificity:** The proportion of true negatives among all actual negative cases, assessing the model’s ability to identify fields without disease.

#### Disease simulation and comparison of outbreak notifications

An algorithm was set to run the wheat blast model for each field sampling location and date using the nearest weather station in Bangladesh and Brazil. Weather data were input into the model, which was set to run continuously from October 1, 2018 forward. The simulation period from November 1 to April 5 covered the 2018–2019, 2019–2020, and 2021–2022 growing seasons in Bangladesh, during which most farmers sow and harvest wheat (Krupnik et al., 2015a, Krupnik et al., 2015b). In Brazil, the model was set to run continuously for each uniform fungicide trial location starting 90 days before sowing in 2016, 2017, 2018, 2019, 2021, and 2022. To evaluate the risk of wheat blast outbreaks, only notifications during the critically sensitive heading stage were included. For both countries, disease risk warnings were generated only during the wheat heading stage based on computed atmospheric spore density (Section 2.3.1). Using conservative thresholds, the model categorizes spore density as high (>10,000 m^−3^), moderate (>4000 m^−3^ and <10,000 m^−3^), or low (<4000 m^−3^).

### Hindcasting spore cloud density and infection

In Bangladesh’s first wheat blast epidemic in 2016, infections affected >15,000 ha, sometimes causing total crop loss (Islam et al., 2019, Malaker et al., 2016). While subsequent outbreaks have been minor, model performance was tested using observed weather data from Jashore, the nearest available weather station to the original outbreak epicenter (Malaker et al., 2016). Historical weather data were used to validate wheat blast risk by simulating days favoring infection and spore cloud density during the wheat season over seven growing seasons (2015–2016 to 2021–2022). A similar hindcasting approach was applied to Uberaba (Brazil) for seven growing seasons (2016–2022). Daily hindcasted spore counts from January 25 to March 5 in Jashore and April 25 to June 1 in Uberaba were used to calculate the area under the spore cloud curve.

## Results

### The scoping stage

Wallach and Scholz (2012)’s framework for UCD begins with identifying key stakeholders involved in the design, evaluation, and use of decision support software, such as the EWS (Fig. 1). In Bangladesh, the BMD, BWMRI, and BARI were identified by CIMMYT as critical for design and evaluation. The DAE, with over field-level 10,000 extension officers, was identified as the primary end-user of the EWS responsible for informing farmers of disease outbreak risks. In Brazil, key stakeholders included EMBRAPA and the *Universidade de Passo Fundo* (UPF), which had already initiated research on wheat blast modeling prior to the 2016 outbreak in Bangladesh. The *Central Cooperativa Gaúcha Ltda*. (CCGL), one of Brazil’s largest farmer cooperatives with over 171 thousand producers, had already expressed interest to UPF and EMBRAPA in the wheat blast model and its potential for advising its farmers, and was therefore included as a relevant extension organization.

Following identification of EWS stakeholders, the scoping stage included a stakeholder workshop convened on July 26–27, 2016 on ‘Mitigating the Threat of Wheat Blast in Bangladesh and Beyond’ (Singh and Barma, 2019), in which options for a EWS in both countries were discussed based on presentation of preliminary modeling results in Brazil reported by Fernandes et al. (2017). Attendance by Bangladesh’s Secretary of Agriculture and subsequent debriefing to the Minister of Agriculture resulted in a request made to CIMMYT to leverage its institutional relationship with EMBRAPA and BMD to develop an EWS for wheat blast. The workshop also resulted in the formation of a National Wheat Blast Task Force comprised of governmental and international agencies involved in plant disease research and extension in Bangladesh. This task force met twice a year, prior to and following the wheat growing season in Bangladesh from 2016 to 2020, and was attended by senior agricultural researchers, the DAE and policy makers. It was during this period which the development of the EWS, its validation, and refinement were standing agenda topics. In Brazil, scientists from EMBRAPA and UPF, who had initiated work on the wheat blast model (Fernandes et al., 2017) collaborated with researchers from the Bangladesh Task Force to embed the model in an operational EWS for use in Brazil. The *Instituto Nacional de Meteorologia* (INMET) was identified by EMBRAPA as a key stakeholder responsible for the supply and scientific use of observed weather data in model validation processes. The process of EWS development in Brazil also included periodic validation and iterative improvement meetings between EMBRAPA, UPF and CCGL to develop systems by which wheat blast advisories could be disseminated to farmers at scale.

### The data collection and analysis stage

Between 2017 and 2019, we conducted key informant interactions, focus groups with farmers, and workshops with agricultural researchers, extension organizations, in Bangladesh and Brazil. Focus group discussions placed emphasis on understanding stakeholder interests as software end-users and how the EWS would function in practice. Key themes from this qualitative data are summarized in Table 1. In both countries, agricultural research institutions (BWMRI, BARI, and the National Task Force in Bangladesh; EMBRAPA and UPF in Brazil) prioritized generating scientific evidence and contributing to model development and evaluation to inform policy and farmer decision-making. In Bangladesh, extension services (DAE) focused on meeting government mandates for an operational EWS and strengthening field officers’ ability to deliver timely disease advisories, while the Bangladesh Meteorological Department (BMD) prioritized the provision of accurate weather data. Interactions with Bangladeshi farmers highlighted their desire for timely, actionable advisories for managing wheat blast. Similarly, Brazilian research institutions (EMBRAPA and UPF) focused on developing scientific tools to support farmers. CCGL emphasized the rapid delivery of weather-based advisories following EMBRAPA’s approval, while the INMET focused on supplying weather data and ensuring its proper use in model evaluation.

**Table 1:** Stakeholder organizations included in the user centered design of the wheat blast Early Warning System (EWS) and interests and roles in EWS design and validation.

Country	Stakeholder type	Stakeholder name	Key interests in the EWS	Preferences for and role in EWS design	Role in EWS validation	Suggestions for advisory content and dissemination
Bangladesh	Agricultural research	Bangladesh Wheat and Maize Improvement Center (BWMRI)	• Fulfillment of the Ministry of Agriculture’s request for an operational wheat blast EWS • Delivery of evidence-based advisories to farmers in alignment with BWMRI mandates • Development of scientific tools to support agricultural policy and planning decisions	• Design and administration of data collection • Refinement and improvement of the model • An API with an interactive dashboard design that can be used for model testing and further research • Development of advisory content • User training and capacity building	• Expert pathologists’ engagement in designing research, running the model, and approving model results and location-specific forecasts	• Advice on safe fungicide application practices • Inclusion of advice on disease resistant cultivars • Inclusion of BWMRI as a source of advice for farmers in emails and SMS advisories
		Bangladesh Agricultural Research Institute (BARI)	• Ongoing interest in wheat research as a crop of interest^b^ • Development of new technology to support farmers	• Provision of advice on field sampling design to assess wheat blast incidence for model validation • Dashboard and map design inputs • Advisory design	• Expert pathologists’ engagement in designing research, running the model, and approving model results and location-specific forecasts	• Advice on safe fungicide application practices

	Extension Organization	Department of Agricultural Extension (DAE)	• Fulfilling the Ministry of Agriculture’s request for an operational wheat blast EWS • Enhancing field officers’ capacity to deliver timely advice on a new disease	• Dashboard and map design inputs, particularly for both tabular and graphical data presentation • Advisory design and dissemination structures aligned with DAE policies • Leading EWS user trainings • Design complementary to ongoing agro-meteorological extension projects	• Supported EWS endorsement following approvals by BWMRI and BARI	• Minimum 5 day lead time • Inclusion of DAE as a source of advice for farmers in emails and SMS advisories • Posting early warnings to the BAMIS portal and in BAMIS bulletins^c^

	Meteorological Agency	Bangladesh Meteorological Department (BMD)	• Institutional mandates to ensure use of observed and forecasted BMD weather data	• Weather data provision and suggestions on weather data use • Model refinement suggestions • Dashboard and map design	• Input into research design • Approving model results and location-specific forecasts	• Use of gridded, high-skill five-day Weather Research Forecasting model forecasts • Linking the EWS to BMD’s website^d^

	Task Force	National Wheat Blast Task Force	• Fulfillment of the Ministry request for an operational wheat blast EWS	• Dashboard and map design inputs • Advisory design and dissemination inputs	• Leading the overall validation and approval process	• Endorsement of BWMRI, BARI, and DAE suggestions

	Farmers	Lead farmers^a^	• Increased advisory support to manage diseases	• Provided suggestions on minimum lead time for forecasts and advisory content	• Supported field data collection for validation • Validated advisory content	• Minimum five-day advisory lead time • Advice on effective fungicide application

Brazil	Agricultural research	*Empresa Brasileira de Pesquisa Agropecuária* (EMBRAPA)	• Delivery of evidence-based advisories to farmers in alignment with EMBRAPA mandates • Development of scientific products to support agricultural policy • Creation of new technologies to assist farmers	• Data collection design and administration • Model refinement and improvement • Dashboard and map design inputs • Design of an API that can be used for future research and model simulations • Advisory design • User training	• Expert pathologists’ engagement in designing research, running the model, and approving model results and location-specific forecasts	• General advice on wheat blast management • Guidance on selecting and using fungicides • Recommendations for disease-resistant cultivars • Advice on wheat blast management tailored to regional wheat-growing areas • Minimum 5 day lead time

		University Passo Fundo (UPF)	• Generation of a scientific • product supporting agricultural policy • Training of students in model application	• Early model development • Model refinement and improvement • Interactive dashboard design supporting research and extension objectives	• Supported early model runs and refinement	• General advice on wheat blast management

	Meteorological Agency	*Instituto Nacional de Meteorologia* (INMET)	• Use of data from its synoptic weather station network	• Weather data provision and suggestions on weather data use	• Validation that observed weather data were used correctly	• Use of gridded, high-skill five-day Weather Research Forecasting model forecasts

	Extension Organization	*Central Cooperativa Gaúcha Ltda*. (CCGL)	• Delivery of timely early warnings to farmer cooperative members based on weather forecasts	• Dashboard and map design inputs • An open-access API capable of supporting data and forecast integration with existing CCGL digital and decision support tools • Simple and flexible advisory design that can be customized • Leading user trainings among coop members	• Led the overall validation and approval process following EMBRAPA’s release of the model • Cooperative lead farmers validated advisory content	• Use of the SmartCoop web application • Farmer-suggested complementary use of WhatsApp, SMS, social media and press-releases for advisory dissemination

a Lead farmers are typically recognized as innovative farmers designated by DAE officers to disseminate information on improved agricultural practices and facilitate knowledge sharing among their village peers.

b Between 1984 and 2017, BARI housed of Bangladesh’s Wheat Research Center. In 2017, Bangladesh’s parliament enacted legislation creating BWMRI as a standalone institution dedicated to wheat and maize.

c Managed by DAE, the Bangladesh Agro-meteorological Information Service (https://www.bamis.gov.bd/en//) produces national- and district-level agrometeorological advisory bulletins issued biweekly to about 30,000 lead farmers.

d The EWS is housed under BMD’s AgroMet section on its website (https://live8.4bmd.gov.bd//).

### The system design and validation stages of user-centered design

The wheat blast EWS was developed through a UCD process that integrated specific stakeholder preferences in Bangladesh and Brazil. The cyclical and interconnected design and validation stages involved iterative development and refinement of both back-end and front-end EWS components, guided by stakeholders’ design preferences and quantitative model validation (Fig. 1). In Bangladesh, DAE prioritized timely, accessible advisories for field officers, leading to the integration of government-approved dissemination channels (Table 1). BMD focused on incorporating accurate five-day WRF weather forecasts to improve disease prediction, while BWMRI and BARI emphasized the need for scientific rigor, contributing to model refinement and interactive dashboard design. In Brazil, CCGL favored rapid, large-scale advisory dissemination via multiple electronic media channels, and EMBRAPA emphasized using high-quality weather data that were supported by model hindcast validation runs. The subsequent sections details how stakeholder-collected weather and disease incidence data supported model validation and performance improvement through hindcasting, critical steps before advancing to the UCD delivery stage.

#### Wheat blast observations

**Fig. 5:**
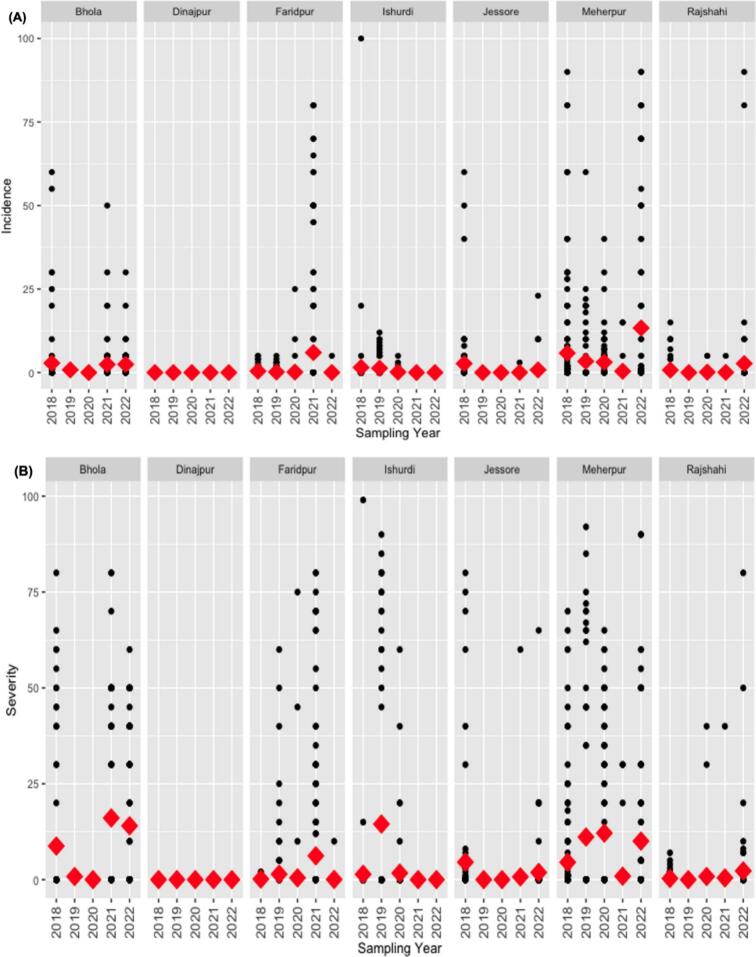
(A) Observed wheat blast incidence and (B) severity (black dots) in wheat fields visited by District at the heading stage in different locations and years in Bangladesh. Red diamonds represent the overall mean for the location and year.

Five years of field surveys led by BWMRI across seven locations in Bangladesh revealed low mean wheat blast incidence and severity, from 0 % to 100 % (Fig. 5A and 5B). In Dinajpur District, wheat blast was absent in all surveys, whereas higher disease frequency was observed in Meherpur, the epicenter of the 2016 outbreak (Malaker et al., 2016). Secondary data from the DAE indicated that only the 2015–2016 wheat season experienced epidemic conditions, while the 2016–2017, 2017–2018, and 2018–2019 seasons had light disease incidence. DAE reported localized infections in select areas during the 2019–2020 season, with subsequent seasons showing relatively light incidence (Supplementary Materials 1). In Brazil, the incidence of wheat blast symptoms on spikes in uniform fungicide trials implemented by EMBRAPA ranged from 0 % to 100 %, with a mean incidence of 49.7 % across 35 trials conducted over different years and locations (Ascari et al., 2021).

#### Observed weather conditions

Due to the absence of weather station data for Meherpur, the epicenter of the initial wheat blast outbreak in Bangladesh in 2016 (Malaker et al., 2016), the model dashboard was used to run continuous disease simulations using data from Jashore, the nearest weather station located approximately 75 km away. Available BMD data from Jashore span from November 1, 2015, to April 15, 2022. During this period, Jashore typically experienced mild, dry weather with lower rainfall compared to other times of the year (Table 2). Across the 2015–2016 to 2021–2022 growing seasons, temperature and relative humidity showed low interannual variability, ranging from 19.9–31.9 °C and 47.4–94.1 %, respectively. In contrast, precipitation varied significantly, from 0 mm in the 2020–2021 season to 332 mm in the 2018–2019 season.

**Table 2:** Observed Weather variables recorded at Jashore, Bangladesh and Uberaba, Brazil during different wheat growing seasons.

Location	Growing season	Temperature (°C)		Relative humidity (%)		Precipitation (mm)
		Maximum	Minimum	Maximum	Minimum	
Bangladesh	2015–2016	31.9	20.8	94.0	50.5	93
	2016–2017	30.9	20.2	93.0	47.9	24
	2017–2018	30.8	20.4	94.1	49.0	189
	2018–2019	31.3	20.3	93.2	47.7	332
	2019–2020	30.7	20.8	92.7	50.4	7
	2020–2021	30.9	19.9	91.4	47.4	0
	2021–2022	30.7	20.4	90.8	50.0	238

Brazil	2016	29.5	17.2	91.3	42.0	175
	2017	29.4	17.6	93.0	45.0	38
	2018	29.6	17.0	90.4	39.9	16
	2019	29.6	17.5	92.0	45.1	398
	2020	28.9	17.0	89.5	42.3	9
	2021	29.4	16.2	89.7	39.2	11
	2022	29.0	16.8	88.1	42.0	11

In Brazil, we Uberaba INMET weather stations and dates from December 1st, 2015 to June 15th, 2022 were selected for model runs. In Uberaba, in Minas Gerais, Brazil, the autumn weather typically starts in March and lasts until May. During this time, the temperatures range from mild to warm, with occasional cool and rainy days. Average temperature in autumn is around 23 °C. During the growing seasons of 2016 through 2022, weather data also suggest low interannual variability in temperature and relative humidity. Temperature and relative humidity ranged from 16.2–29.6 °C and 39.2–93.0 %, respectively. Rain, however, varied from 9 (2020) to 398 mm (2019).

#### Hindcasting comparison of field observations with model-generated outbreak notifications

For each of the 304 fields sampled by BWMRI in Bangladesh, CIMMYT, BWMRI and BARI researchers agreed to run the model using seasonal weather data from the nearest weather station. A predicted outbreak was assigned a value of 1 if five or more alerts occurred during the heading stage; otherwise, it was set to 0. Similarly, actual field data were set to 1 (outbreak) if the mean wheat blast incidence exceeded 20 %; otherwise, they were set to 0. Correct and incorrect predictions were summarized by class in a confusion matrix (Table 3). Here, binary class classifications in rows are represented by 0 (non-epidemic) and 1 (epidemic), while data presented diagonally across columns represent correct predictions. Non-diagonal data suggest the opposite.

**Table 3:** Confusion matrices of the predicted and actual occurrence of wheat blast in 304 fields visited in different locations and years in Bangladesh and 35 uniform fungicide trials in Brazil.^a^

Bangladesh		Brazil	
No Wheat Blast	Wheat Blast	No Wheat Blast	Wheat Blast
True Negative: 233	False Positive: 20	True Negative: 9	False Positive: 1
False Positive: 43	True Negative: 8	False Positive: 2	True Positive: 23
Classification measures			
Precision	0.84	0.81	
Negative Predictive Value	0.15	0.92	
Accuracy	0.79	0.91	
Sensitivity	0.84	0.81	
Specificity	0.28	0.95	

a See section 2.4.3 for definitions of these metrics.

Comparisons of the wheat blast model with observed data in Bangladesh show relatively high overall accuracy (0.79), though interpretation should be made with caution due to the imbalance in the dataset. Most field observations (253 of 304) fell into the no disease class, and the model correctly predicted the majority of these cases (233 true negatives). However, it failed to identify 43 fields that experienced wheat blast (false negatives), resulting in a low negative predictive value (0.15). This metric, which quantifies the model’s ability to correctly identify true negatives among predicted negatives, is sensitive to class imbalance. In contrast, the model achieved high precision (0.84), reflecting strong performance in predicting positive cases where they were observed. The majority of field-observed wheat blast cases were concentrated in Bhola and Faridpur districts (data not shown), aligning with known spatial patterns of disease incidence. Model runs from 2015 to 2022 confirm the need to interpret predictive metrics in relation to the underlying distribution of disease severity in the dataset.

**Fig. 6:**
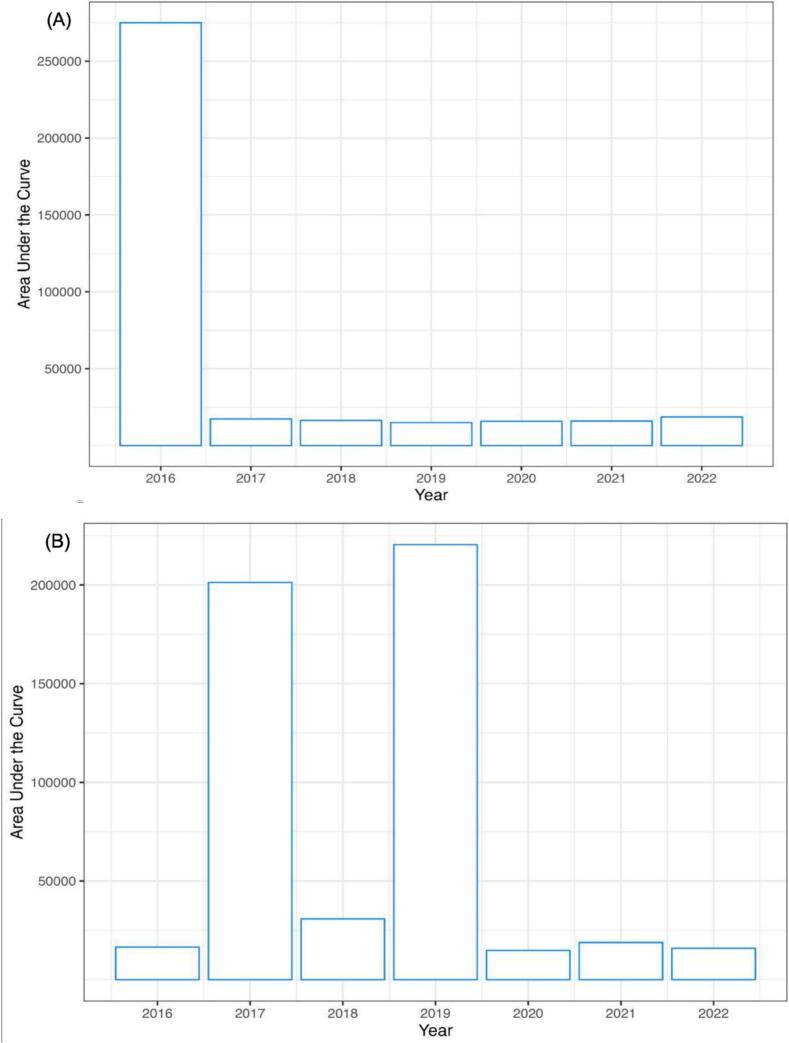
Area under the curve for the simulated daily density of wheat blast conidia over a plausible window of a wheat crop at the wheat heading stage in Jashore, (A) Bangladesh and (B) Uberaba, Brazil. In Bangladesh, the heading period covers January 25th through March 5th, and Brazil, from April 25th through June 5th.

Model performance was also assessed using observed weather data from Jashore, Bangladesh, to hindcast wheat blast risk and analyze EWS-generated disease alert patterns over seven growing seasons (2015/2016–2021/2022). The model triggers EWS alerts based on spore density in 1 m^3^ of air above the crop canopy (Section 2.3.1). Simulations from November 1, 2015, to March 5, 2016—the season of Bangladesh’s first wheat blast outbreak—showed that spore cloud density, influenced by temperature, relative humidity, and precipitation, could infect wheat spikes. Supplementary Materials 3 details daily simulated spore densities alongside temperature, relative humidity, and precipitation data for the 2015–2016 to 2021–2022 wheat seasons in Jashore. In contrast, spore densities in subsequent seasons, represented under the density curve, were much lower (Fig. 6A and B), aligning with DAE data indicating outbreak conditions only in 2015–2016 and limited disease incidence or localized infections in later years (Supplementary Materials 2).

In contrast, model performance in Brazil, tested by EMBRAPA and UPF researchers using observed data, was consistent across all metrics. In Uberaba, located in the Cerrados, wheat blast outbreaks occurred in 2017 and 2019, with 2019 being one of the most severe outbreaks recorded (Santana et al., 2020, EMBRAPA, 2019). Model hindcasting indicated high spore cloud density in those years, driven by favorable temperature, relative humidity, and rainfall conditions (Supplementary Materials 4). In contrast, dry growing seasons in 2020, 2021, and 2022 resulted in minimal wheat blast occurrence.

### The delivery stage of user-centered design

In the final stage of UCD, stakeholders were engaged in designing written disease advisories, disease risk maps, and dissemination pathways (Table 1). Interactions with stakeholder organizations also focused on preparing them for EWS use and advisory interpretation through structured training sessions and workshops conducted in Bangladesh and Brazil from 2019 to 2022. Participants included field-level and senior extension experts, farmer cooperative members, and agricultural researchers. Usability testing, typically part of UCD validation, was integrated into extension staff training during the delivery phase. This iterative process directly informed improvements in both the software’s back-end and front-end design (Fig. 1).

#### The early warning system

##### Back-end structure

Based on stakeholder feedback, the server-side components of the Wheat Blast EWS included an API designed to process and analyze data from ground-based weather stations and numerical weather forecasts. These data generate wheat blast forecasts displayed through an interactive web interface, informed by stakeholder and user input (Table 1). Weather station data and numerical forecasts were formatted for efficient processing. The server-side infrastructure was built using Node.js (Version 16.8), the Express.js web framework (Version 4.16), and a MongoDB NoSQL database (Version 4.0). A web service, developed in R (Version 3.6.3; R Core Team, 2022) with the Plumber package (Version 1.2; Schloerke and Allen, 2022), managed model execution and data processing, with system components communicating via JSON message exchange.

##### Front-end structure

Based on key informant interactions and focus groups conducted during the 2017–2019 design and validation UCD stages in Bangladesh and Brazil, the EWS client-side components were developed. Researchers and extension officials emphasized the need for an interactive, dynamic user interface that allows customization of inputs for specific locations, weather conditions, and simulation date ranges (Table 1). In response, a web interface, interactive maps, graphs, and a rapid disease risk alert notification system (Section 2.4.3) were created. The web interface used Embedded JavaScript templates (EJS) to generate HTML pages from JavaScript (V. ES6, San Francisco). CSS and Bootstrap were used for styling, while Leaflet (V. 0.7) was used to generate interactive maps. Plotly.js (V. 1.58.5, Montreal, Canada) was integrated to produce a dynamic, responsive web application for disease risk updates in line with users’ preferences.

A dashboard built with Node.js and Embedded JavaScript templates was implemented to display historical and real-time weather information in a web application (https://beattheblastews.net/) developed using the R Package Shiny (Version 1.7.4). The dashboard allows users to select the country, location, weather station, and date to initiate simulations and to project future wheat blast outbreak risks. Resulting data are organized into tabs displaying wheat blast risk levels—low, moderate, and high. Based on user preferences, and particularly the DAE, the dashboard also provides graphs of weather data used in simulations and model validation, along with combined model outputs and weather data in tabular format (Table 1).

##### Alert system structure

**Fig. 7:**
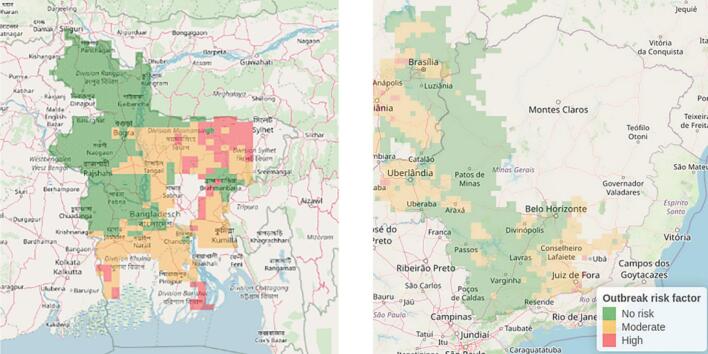
Model output maps from the disease early warning system in Bangladesh (left) and Brazil’s Cerrados region (right) are shown on the online dashboard. Green, orange, and red grid cells indicate no, moderate, and high risk, respectively, as described in Section 2.4.3. Map gaps reflect areas with little or no wheat cultivation.

The EWS’s alert system structures integrates WRF Model forecasts (Version 4.4.2, Boulder, USA) from the Bangladesh Meteorological Department at a 17 × 17 km grid resolution for all wheat-growing regions. In Brazil, the IBM Weather Company supplies numerical weather forecasts at a 10 × 10 km resolution, which CCGL uses to provide wheat blast risk forecasts to registered farmers and extension staff. Based on consultations with BMD in Bangladesh and EMBRAPA in Brazil, and confirmation by CCGL and DAE, gridded forecasts are generated hourly on a rolling basis for five days—the maximum forecast window deemed reliable by the meteorological organizations involved in EWS design felt reliable for issuing agricultural advisories (Table 1). This timeframe, agreed upon by BMD, DAE, and EMBRAPA, balances forecast accuracy with a lead time that the latter two stakeholders felt sufficient for farmers to act. For each grid cell with processed weather data, the model generates maps of wheat-growing areas in Bangladesh or specific regions in Brazil, displaying daily wheat blast risk forecasts on the EWS dashboard for the five-day forecast period (Fig. 7).

While researchers and extension officials in both countries prioritized an interactive web-based dashboard, their dissemination preferences differed (Table 1). In Brazil, CCGL preferred use of the SmartCoop application (https://app.smart.coop.br/) to deliver wheat blast risk advisories, supplemented by SMS, WhatsApp groups for cooperative members, and use of press releases and social media (Fig. 1). In contrast, Bangladesh’s DAE distributed advisories through bi-weekly agro-meteorological bulletins and sent pre-structured email and SMS alerts to extension officers and lead farmers during outbreak risks (Table 4), along with training extension agents on the EWS dashboard.

**Table 4:** The structure of email and SMS messages from the Wheat Blast Early Warning System in Bangladesh is based on the wheat crop’s risk level and development stage. Advisories are sent only to registered users in locations where moderate or high-risk conditions are forecasted during wheat heading, flowering, and ripening stages.

Forecast risk level	Phenological stages and potential date range corresponding to this stage			
	9 Jan–18 Feb Heading stage	19 Jan–28 Feb Flowering stage	29 Jan–10 March Ripening stage	23 Feb–4 April Maturity stage
No risk	No risk of disease outbreak. No advisory is provided			
Moderate risk	Current weather conditions favor wheat blast disease infection. If wheat is close to or at heading, then you may wish to consult DAE, BWMRI, or CIMMYT and apply fungicide in the next few days to prevent infection. Fungicides should only be applied using appropriate health and safety equipment and precautions.	Current weather conditions favor wheat blast disease infection. If wheat is at heading or flowering and you may wish to consult DAE, BWMRI, or CIMMYT and apply fungicide in the next few days to prevent infection. Fungicides should only be applied using appropriate health and safety equipment and precautions.	Current weather favor wheat blast disease infection. If wheat is at flowering, then you may wish to consult DAE, BWMRI, or CIMMYT and apply fungicide in the next few days to prevent infection. Fungicides should only be applied using appropriate health and safety equipment and precautions.	No risk of disease outbreak. No advisory is provided
High risk	Current weather conditions favor wheat blast disease infection. If wheat is close to or at heading, then you may wish to consult DAE, BWMRI, or CIMMYT and apply fungicide immediately to avoid risk of disease outbreak. Fungicides should only be applied using appropriate health and safety equipment and precautions.	Current weather conditions favor wheat blast disease infection. If wheat is at heading or flowering, then you may wish to consult DAE, BWMRI, or CIMMYT and apply fungicide immediately to avoid risk of disease outbreak. Fungicides should only be applied using appropriate health and safety equipment and precautions.	Current weather conditions favor wheat blast disease infection. If wheat is at flowering or ripening, then you may wish to consult DAE, BWMRI, or CIMMYT and apply fungicide immediately to avoid risk of disease outbreak. Fungicides should only be applied using appropriate health and safety equipment and precautions.	As the wheat crop is likely to be largely mature, the risk of disease outbreak. No advisory is emailed or provided

## Discussion

Pequeno et al. (2024) projected that increasing warm and humid conditions driven by climate change could lead to a 69 million ton annual reduction in wheat production due to wheat blast by mid-century, particularly in the Southern Hemisphere. Wheat blast management typically involves disease-resistant cultivars, farmers adjusting their sowing dates and ensuring use of healthy seeds, and fungicide applications. However, applying agricultural climate services to assess location-specific disease risks and disseminate management advisories remains an emerging research field (Charalampopoulos and Droulia, 2024, Mason et al., 2022, Matengu et al., 2023, Smith et al., 2024). This study utilized a user-centered software design framework (Wallach and Scholz, 2012) to develop a weather forecast-driven early warning system (EWS) for wheat blast in Brazil and Bangladesh. These countries were the first to report the disease in South America and South Asia, respectively, where blast poses a significant threat to wheat production and where agricultural research and extension organizations have a history of collaboration (Fernandes et al., 2019).

This study applied a user-centered software design framework (Wallach and Scholz, 2012) to develop a weather-forecast-driven early warning system (EWS) for wheat blast in Bangladesh and Brazil, where the disease threatens wheat production and agricultural research and extension organizations have a history of collaboration (Fernandes et al., 2019). The co-design process, based on qualitative data collection and thematic analysis (Braun and Clarke, 2006), involved scoping, analysis, system design, and delivery stages that facilitated stakeholder engagement for iterative system development, validation, and refinement. In Bangladesh, stakeholder engagement was reinforced by a Ministry of Agriculture directive supporting the development and release of an EWS for wheat blast (Singh and Barma, 2019). User feedback during the data collection, analysis, and the system design stages guided the server-side development of the weather-forecast-driven disease model and its API, informing decisions on model parameters, data integration, and user interface design. Agricultural researchers from BWMRI, BARI, and BMD in Bangladesh and EMBRAPA, INMET, and UPF in Brazil contributed to research co-design, data collection, model validation, data interpretation, and performance assessment. Meteorological agencies (BMD, INMET, and EMBRAPA) provided observed and forecasted data on temperature, relative humidity, and precipitation to support model operations and advised on data processing and visualization for hindcasting validation and disease forecasting. Feedback from DAE in Bangladesh and CCGL in Brazil emphasized the need for timely, localized advisories, shaping the API’s structure to generate and deliver targeted risk notifications. The co-design process aligned the system’s development with the operational needs of agricultural researchers, extension agents, meteorological services, and farmer cooperatives. Validation in Bangladesh involved expert reviews by BWMRI, BARI, and the National Wheat Blast Task Force, with input from DAE. In Brazil, EMBRAPA, UPF, CCGL, and INMET validated the system through iterative research.

Results from the UCD design and validation stages demonstrate the influence of temporal and spatial variability on disease pressure, as observed in field surveys in Bangladesh and uniform fungicide trials in Brazil. Model hindcast testing produced satisfactory results in Jashore, Bangladesh, and Uberaba, Brazil. In Bangladesh, warm and humid conditions early in the wheat-growing season encourage wheat blast development (Supplementary Materials 3), while cooler temperatures and reduced precipitation later in the season limit pathogen growth. However, during the 2016 outbreak (Islam et al., 2019, Malaker et al., 2016), minimum temperatures in Jashore remained above 20 °C during the grain-filling stage. Nighttime temperatures above 20 °C also accelerate crop senescence (Prasad et al., 2008). Cruz et al. (2015) reported that MoT causes more lesions on older leaves. Accelerated aging processes are associated with higher temperatures, and can weaken wheat plants, increasing vulnerability to infection and enabling fungal growth and spore production under favorable conditions.

Over five years of surveys across seven locations in Bangladesh, wheat blast incidence and severity remained low. Hindcast runs from 2015 to 2022 yielded precision and negative predictive values of 0.84 and 0.15, respectively. This suggests the model effectively predicts disease occurrence, allowing farmers to implement preventative measures. These values reflect the model’s assumption of a uniform environmental presence of inoculum to simulate conidia and lesion cohort development, excluding source-sink dynamics and spore dispersal, which future research should explore (Smith et al., 2024). The 2016 epidemic in Bangladesh caused losses exceeding 15,000 ha (Islam et al., 2019, Malaker et al., 2016), but no major outbreaks have occurred since. However, field surveillance and extension reports indicate sporadic, localized and sometimes severe infections (Supplementary Materials 2), suggesting an uneven MoT presence in wheat-growing regions (Supplementary Materials 3). Seasonal diurnal temperature ranges (DTR) in Bangladesh generally decrease during the winter and pre-monsoon periods (Shahid et al., 2012), but exceptionally low DTR was observed during the 2015–2016 growing season (Supplementary Materials 3), possibly due to cloud cover reducing UV exposure and trapping heat near the ground (Hamal et al., 2021). Lower DTRs have been linked to dew formation and high-humidity microclimates that favor disease development (Lobell 2007). Variability in disease occurrence in Bangladesh may also result from cultivar susceptibility, contaminated seeds, and microclimatic conditions (Fernandes et al., 2017, Fernandes et al., 2021, Juliana et al., 2022, Malaker et al., 2016, Montes et al., 2022), which should inform future model and EWS improvements.

In Brazil, where wheat blast has been established for over 30 years (Fernandes et al., 2017), inoculum distribution is likely more uniform than in Bangladesh. Model validation used wheat blast incidence data from control plots in uniform fungicide trials in disease hotspots with susceptible cultivars (dos Santos et al., 2022), with confusion matrix analysis showing solid model performance. Warm, humid conditions at the start of the wheat-growing season in the Cerrados favor disease development (Fernandes et al., 2017), but fewer rain events and lower humidity later in the season appear to reduce sporulation. Model runs for Uberaba, Brazil, in 2017 and 2019 indicated weather conditions conducive to spore cloud densities capable of causing infections (Supplementary Materials 4). Both years corresponded with reported wheat blast epidemics in Brazil (Ascari et al., 2021).

During the delivery stage of UCD, collaboration with extension organizations in both countries supported the co-design of advisory content and dissemination strategies, coupled with stakeholder training to improve system usability, scaling and impact. In Bangladesh, DAE prioritized SMS notifications, email alerts, and weekly agro-meteorological bulletins via the Bangladesh Agro-Meteorological Information System (BAMIS) portal. The EWS dashboard and advisories were also made accessible through the DAE and BMD websites. DAE staff and lead farmers were trained to interpret and share advisories. In Brazil, while CCGL accesses the dashboard, they preferred use of the underlying API to make use of their own SmartCoop web application for advisories, supplemented by WhatsApp groups, SMS alerts, social media posts, and press releases. This multi-channel strategy reflected stakeholder feedback and preferences for diverse dissemination channels. The distinct stakeholder roles and preferences in Bangladesh and Brazil underscore the need to adapt system design, dissemination, and scaling strategies to different cultures, institutional frameworks, and capacities.

This co-development process led to the formal endorsement of the EWS by Bangladesh’s Ministry of Agriculture in December 2019 and by CCGL in 2021. To date, 8,576 DAE staff and farmer group leaders in Bangladesh have been trained to interpret advisories and receive real-time disease risk updates. In Brazil, about 5,000 farmers and 1,000 field agronomists in southern wheat-growing regions receive mobile and WhatsApp notifications from the EWS API, integrated into SmartCoop, with advisories customized by CCGL’s technical division. When the EWS detects infection risk, CCGL issues tailored alerts via press releases, social media, and SMS, providing timely guidance to wheat growers and crop advisers.

While the UCD process and wheat blast EWS show potential, several research and practical limitations should be addressed. Firstly, the UCD process focused primarily on extension organizations—DAE in Bangladesh and CCGL in Brazil—as ultimate end-users. Future research should incorporate more feedback from farmers to evaluate advisory use and improve content, structure, and dissemination. Secondly, examining how and when extension officers communicate warnings could guide further system enhancements and improve approaches to scale-out use of the EWS. Exploring longer lead times beyond five-day forecasts may offer farmers more time for preventive action. Thirdly, although the choice of a 5-day weather forecast to drive the EWS was influenced by stakeholders, particularly BMD in Bangladesh and EMBRAPA in Brazil, longer forecast periods could be useful though thorough examination of forecast accuracy would be needed. This topic, while relevant, is beyond the scope of the current study that focuses on UCD of EWS design. Furthermore, research indicates that sub-seasonal forecast accuracy remains limited in Bangladesh (Mason et al., 2022); future research should therefore assess trade-offs between forecast skill, model performance, and farmers’ response. The model could also be tested in Bolivia, Paraguay, Argentina (Fernandes et al., 2017, Fernandes et al., 2021), in and Zambia where wheat blast was most recently observed (Tembo et al., 2020), with stakeholder engagement supporting system expansion through the API where performance is adequate.

## Conclusions

This study described the development of a climate service using a UCD framework to design a weather-driven EWS for managing wheat blast in Brazil and Bangladesh. By integrating real-time and forecasted weather data—specifically daily cardinal temperature, relative humidity, and accumulated rainfall—from national meteorological services, the EWS translates complex weather information into actionable, location-specific advisories. The UCD process, which included stakeholder scoping, data analysis, system design, and delivery, aligned the EWS with the operational needs of extension services. Iterative model development and validation using empirical disease data, combined with active stakeholder engagement in cross-learning settings, enhanced system reliability and relevance. Model evaluations showed strong predictive performance, with EWS alerts matching observed wheat blast outbreaks. Hindcasting analyses of spore cloud density across seven wheat seasons further confirmed the model’s capacity to capture climate-driven disease dynamics, reinforcing its value as a climate-informed risk management tool.

The climate service was co-developed using a mixed methods approach that required interdisciplinary collaboration among agricultural research institutes, meteorological agencies, and extension organizations. This enabled formal endorsement of the EWS by government authorities in both countries. Collaboration supported country-specific strategies to interpret model outputs and deliver advisories, illustrating how adaptive climate services can be customized to fit operational needs.

The UCD framework described in this paper offers a flexible method for developing agricultural climate services targeting weather-sensitive crop diseases. Successful application and scaling of advisory systems in other contexts will require ongoing refinement to reflect changing climate conditions and evolving user needs. Future research may consider integrating longer forecast lead times to deliver earlier warnings while balancing accuracy with usability. Enhancing the use of seasonal and sub-seasonal climate forecasts could also strengthen the system’s ability to support proactive, climate-resilient agricultural decisions. By linking climate science with agricultural practice, the EWS for wheat blast described in this paper demonstrates how climate services can reduce vulnerability to plant disease outbreaks and support more sustainable agricultural practices amid increasing climate variability.

## CRediT authorship contribution statement

**Timothy J. Krupnik:** Writing – review & editing, Writing – original draft, Visualization, Software, Methodology, Investigation, Formal analysis, Data curation, Conceptualization. **José Mauricio Cunha Fernandes:** Writing – review & editing, Writing – original draft, Visualization, Software, Methodology, Formal analysis, Data curation, Conceptualization. **Felipe Vargas:** Writing – review & editing, Visualization, Software, Methodology. **Emerson Medeiros Del Ponte:** Writing – review & editing. **Khaled Hossain:** Writing – review & editing, Investigation, Data curation. **Mustafa Kamal:** Writing – review & editing, Software, Data curation. **Mutasim Billah:** Writing – review & editing, Software, Data curation. **Md. Harun-Or-Rashid:** Writing – review & editing, Investigation, Data curation. **Sk. Ghulam Hussain:** Writing – review & editing, Validation, Data curation. **Pawan Kumar Singh:** Writing – review & editing, Validation. **Krishna Kanta Roy:** Writing – review & editing, Validation. **Carlos Augusto Pizolotto:** Writing – review & editing, Validation. **Md. Shah Kamal Khan:** Writing – review & editing, Validation. **Willingthon Pavan:** Writing – review & editing, Validation, Software. **Golam Faruq:** Writing – review & editing, Validation.

## Declaration of competing interest

The authors declare that they have no known competing financial interests or personal relationships that could have appeared to influence the work reported in this paper.

## Data Availability

Data will be made available on request.
