# Supplementary material for: A weather-forecast driven early warning system for wheat blast disease: User-centered design, validation, and scaling in Bangladesh and Brazil
Source: Clim Serv. 2025 Aug;39:100589. doi: 10.1016/j.cliser.2025.100589 (PMC12350828; doi:10.1016/j.cliser.2025.100589)
Supplement: Supplementary Data 1 [file mmc1.docx]

**Supplementary Materials 1**: Pest disease coefficients (COEFF) of the Wheat Blast EWS

COEFF DEFINITIONS

! ===== ===========

! PESTID# Code for the disease used on FileX (PESID under Management section)

! PSTNAME Pest description

! CP Coupling Point Type (VEG,ROOT,ASM,SEED,STEM)

! DSPL Daily Spore Production per Lesion

! SPE Spore Production Efficiency - a set of values used in a trapezoid function based on

cohort age (Min, Opt1, Opt2, Max)

! SCF Sporulation Crowding Factors - a set of values based on the population density

! MSCD Maximun Spore Clouds Density

! ASR Attainable Spore Rate - Proportion of spores produced able to infect (**** Verificar DF)

! SPO2P Spore Proportion that moves from Organ cloud to Plant cloud

! SPP2F Spore Proportion that moves from Plant cloud to Field cloud

! CCFPO Cloud compartimentalization (days till leave the system - die/remove) (Field, Plant,

Organ)

! II Initial Inoculum

! AFII Accumulate Favorability for release of the starting inoculum based on temperature

favorability set (#acumulateFavorability)

! TFS Temperature Favorability Set - cardinal temperatures used to calculate the temperature

favorability in the infection period (maximum, minimum and optimal)

(#temperatureFavorabilitySet)

! IE Infection Efficiency (#infectionEfficiency)

! DF Dispersion Frequency - Proportion of spores (#dispersionFreequency) (*** Verificar

ASR)

! IPS Initial Pustule Size (#initialPustuleSize)

! LP Latent Period - Number of biological days based on TFS (Temperature Favorability Set)

(#latentPeriod)

! IP Infection Period - Number of biological days based on TFS (Temperature Favorability

Set) (#infectionPeriod)

! WT Wetness Threshold - Minimal number of hours necessary with free water available for

infection (#wetnessThreshold)

! HF Host Factor - Resistance factor for the disease by host (0-1; 1 no resistant; 0 resistant)

(#hostFactor)

! IGF Invisible Growth Function - Function that will be evaluated during the latent period

(including parameter values) (#invisibleGrowthFunction)

! VGF Visible Growth Function - Function that will be evaluated during the latent period

(including parameter values) (#visibleGrowthFunction)

! MRRS Millimeters of rain needed to reduce spores cloud number

!

! Wheat Blast set to VEG since the Spore Cloud is being calculated through infected leaves

@PESTID# PSTNAME............. CP DSPL SPE SCF MSCD ASR

SPO2P SPP2F CCFPO II AFII TFS IE DF IPS LP IP WT HF

DRE..................................................... WF........................... IGF..........................

VGF..................................... MRRS RHFac........

!Below Felipe .JSON file (need to define the following variables - "LC":"G","SWF":"1")

WH001 Wheat Blast VEG 1500 6,15,60,100 0.98669,10.71894,0.93374 15000 0.044

0.25 0.45 4,6,8 100 0 30,15,28 0.8 0.15 0.001 7 21 8 1

0.367753*(x+0.001)^0.129605*exp(-0.085252*(x+0.001)) 1/(1+exp(4.948-0.348*x)) 0.01+

(x*0.2/9) 0.21+((x-9)*0.1/21) 2000 1

!Below WB coeff paper Nature

! WH001 Wheat Blast VEG 1500 6,15,22,30 0.98669,10.71894,0.93374 15000 0.044

1 1 3,6,9 500 0 30,15,28 0.8 0.15 0.001 7 21 8 0.7

0.367753*(x+0.001)^0.129605*exp(-0.085252*(x+0.001)) 1/(1+exp(4.948-0.348*x)) 0.01+

(x*0.2/9) 0.0161858*exp(-exp(1.563509-0.441721*x)) 15 1

WH002 Tan Spot VEG 100 1,15,15,20 0.98669,10.71894,0.93374 15000 0.1

0.25 0.45 3,6,9 500 0 32,10,25 0.7 0.15 0.001 8 20 8 1

0.367753*(x+0.001)^0.129605*exp(-0.085252*(x+0.001)) 1/(1+exp(4.948-0.348*x))

0.036*(1.0104-exp(-0.3125*x)) 0.0161858*exp(-exp(1.563509-0.441721*x)) 15 1

WH003 Powdery Mildew VEG 1000 1,15,15,20 0.98669,10.71894,0.93374 15000

0.1 0.25 0.45 3,6,9 500 0 26,5,22 0.7 0.15 0.001 8 20 0 1

0.367753*(x+0.001)^0.129605*exp(-0.085252*(x+0.001)) 1*exp(-2.22*x)

0.036*(1.0104-exp(-0.3125*x)) 0.0161858*exp(-exp(1.563509-0.441721*x)) 15 (-0.3+.018*x)

WH004 FHB SEED 300 6,15,15,20 0.98669,10.71894,0.93374 15000 0.044

0.25 0.45 3,6,9 0 0 30,15,28 0.7 0.15 0.001 10 20 8 0.8

0.367753*(x+0.001)^0.129605*exp(-0.085252*(x+0.001)) 1/(1+exp(4.948-0.348*x)) 0.01+

(x*0.2/9) 0.0161858*exp(-exp(1.563509-0.441721*x)) 15 1

!IE <- Evans, C. K., Hunger, R. M., & Siegerist, W. C. (1996). Inoculum density and infection

efficiency of conidia and conidiophores of isolates of Pyrenophora tritici-repentis. Plant Disease,

80(5), 505�512. https://doi.org/10.1094/PD-80-0505

!DSPL, SPE, MSCD,<-Riaz, M. (1991). Effects of Wheat Genotype, Time After Inoculation, and

Leaf Age on Conidia Production by Drechslera tritici-repentis . Phytopathology, 81(10), 1298.

https://doi.org/10.1094/phyto-81-1298!

!WT<- Effects of Leaf Wetness Duration and Inoculum Level on Resistance of Wheat

Genotypes to Pyrenophora tritici

! If the rainfall during the current hour is over 0.5 mm, then wetness is expected. If there is no

rainfall, the wetness depends on the relative humidity higher than 90%

! during the current hour and the amount of rain or wetness that occurred in the previous hour.

In the absence of rainfall, the difference between the minimum and dew point

! temperature should be less than 3.8 C to expect wetness.

! CCFPO can be affected by a cloudiness factor estimated based on daily temperature range.

! MSCD <- Li, Y. (2013). Factors influencing the development of gray leaf spot of perennial

ryegrass turf and seasonal availability of the inoculum (PhD Thesis). The Pennsylvania State

University.

**Supplementary Materials 2**. Wheat Blast affected areas and districts from 2016 to 2022 in Bangladesh^a^.^.^

| Region | District | Blast affected area (ha) | | | | | | |
| --- | --- | --- | --- | --- | --- | --- | --- | --- |
|  |  | 2015-16 | 2016-17 | 2017-18 | 2018-19 | 2019-20 | 2020-21 | 2021-22 |
| South-West | Jashore | 1.500 | 2 | 0 | 0 | 5 | 0 | 0 |
|  | Jhenaidah | 840 | 1 | 0 | 0 | 43 | 0 | 0 |
|  | Magura | 0 | 1 | 0 | 0 | 0 | 0 | 0 |
|  | Kushtia | 320 | 1 | 0 | 0 | 239 | 0 | 0 |
|  | Chuadanga | 2.563 | 0 | 8 | 0 | 10 | 0 | 0 |
|  | Meherpur | 9.640 | 8 | 1 | 2 | 5 | 0 | 0 |
| Central-West | Rajshahi | 0 | 1 | 4 | 0 | 0 | 0.35 | 0 |
|  | Natore | 0 | 0 | 4 | 0 | 0 | 0.25 | 0.14 |
| Central | Pabna | 50 | 0 | 0 | 0 | 17 | 0.5 | 0 |
|  | Sirajganj | 0 | 0 | 0 | 0 | 0 | 0.34 | 0 |
|  | Faridpur | 0 | 7 | 0 | 0 | 0 | 0 | 0 |
| Southern | Shariatpur | 0 | 0 | 1 | 0 | 2 | 0 | 0 |
|  | Bhola | 500 | 0 | 0 | 0 | 0 | 0 | 0 |
|  | Barishal | 20 | 0 | 0 | 0 | 0 | 0 | 0 |
| Total | | 15.433 | 20 | 16 | 2 | 321 | 1.44 | 0.14 |

^a.^ Source: Department of Agriculture Extension (DAE). Bangladesh 2015-16 data presented by Directors of Field Services (DFS) and Additional Directors (ADs) at the International Consultation workshop at Kathmandu. Nepal (<https://csisa.org/mitigating-wheat-blast-in-bangladesh/>). 2016-17 data were collected from the DFS, DAE, in Bangladesh, in a 2nd follow-up workshop on wheat blast at Banani. Dhaka 2017-18. 2018-19, 2019-20, 2020-21, and 2021-22 data were collected from respective ADs in DAE offices in each district. 2015-16 was considered to be a wheat blast outbreak wheat growing season by DAE. 2016-17, 2017-18, and 2018-19 were considered to be years of light incidence. 2019-20 was considered a year in which localized infections were observed in select locations, while subsequent wheat growing seasons were considered to be characterized by light incidence only.

**Supplementary Materials 3.** Daily simulated conidia density (gray line) with accompanying weather data from the wheat growing seasons of 2015-2016 through 20121-2022 in Jashore, Bangladesh. Note that 2015-16 was the epidemic growing season. The shaded area corresponds approximately to the heading stage window. Daily maximum temperature (red line) and minimum temperature (blue line), daily maximum relative humidity (dark violet line), daily minimum relative humidity (violet line), and daily accumulated rainfall (vertical bars).

| 2015-16 Wheat Growing Season |
| --- |
| 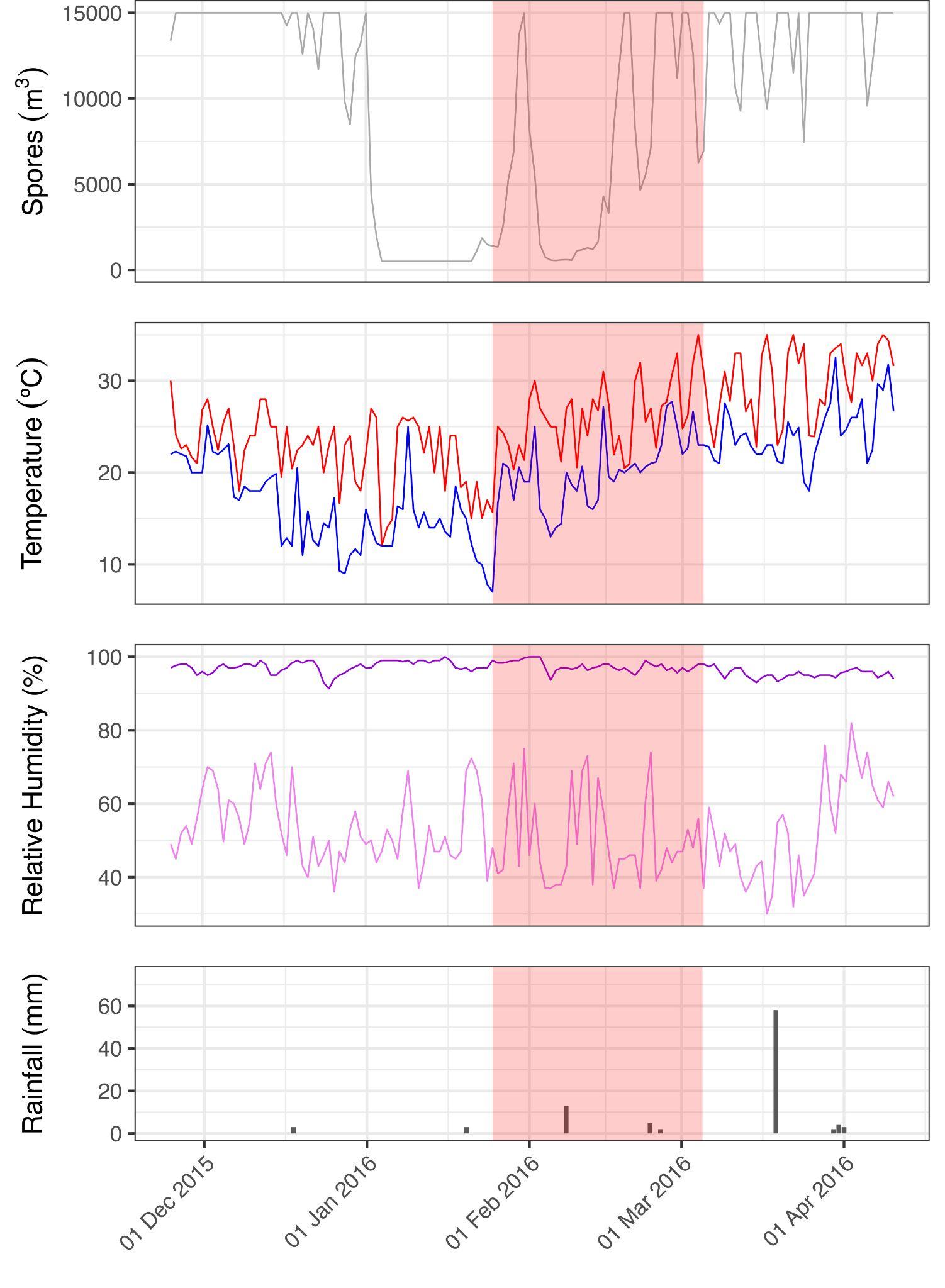 |

| 2016-17 Wheat Growing Season |
| --- |
| 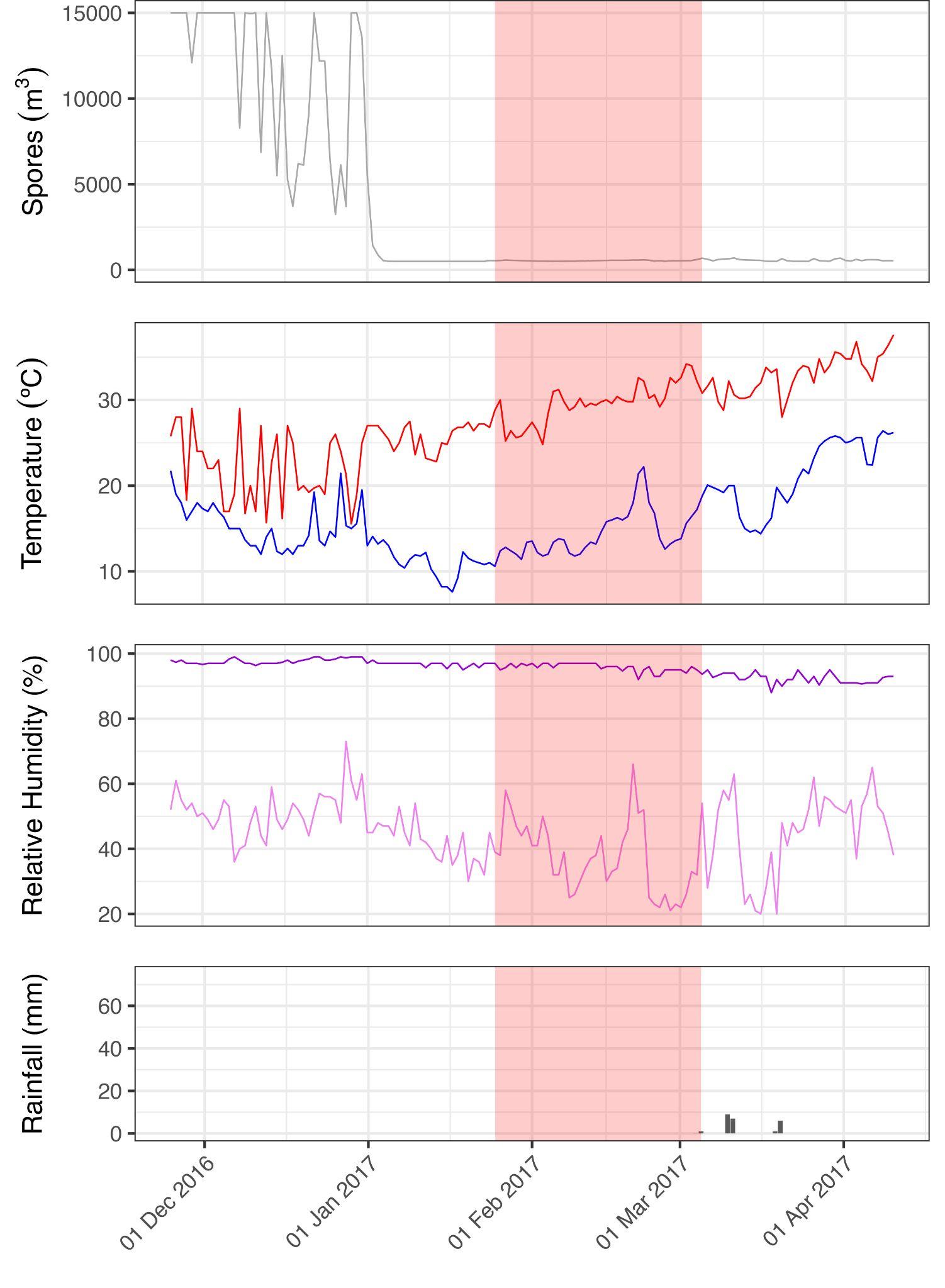 |

| 2017-18 Wheat Growing Season |
| --- |
| 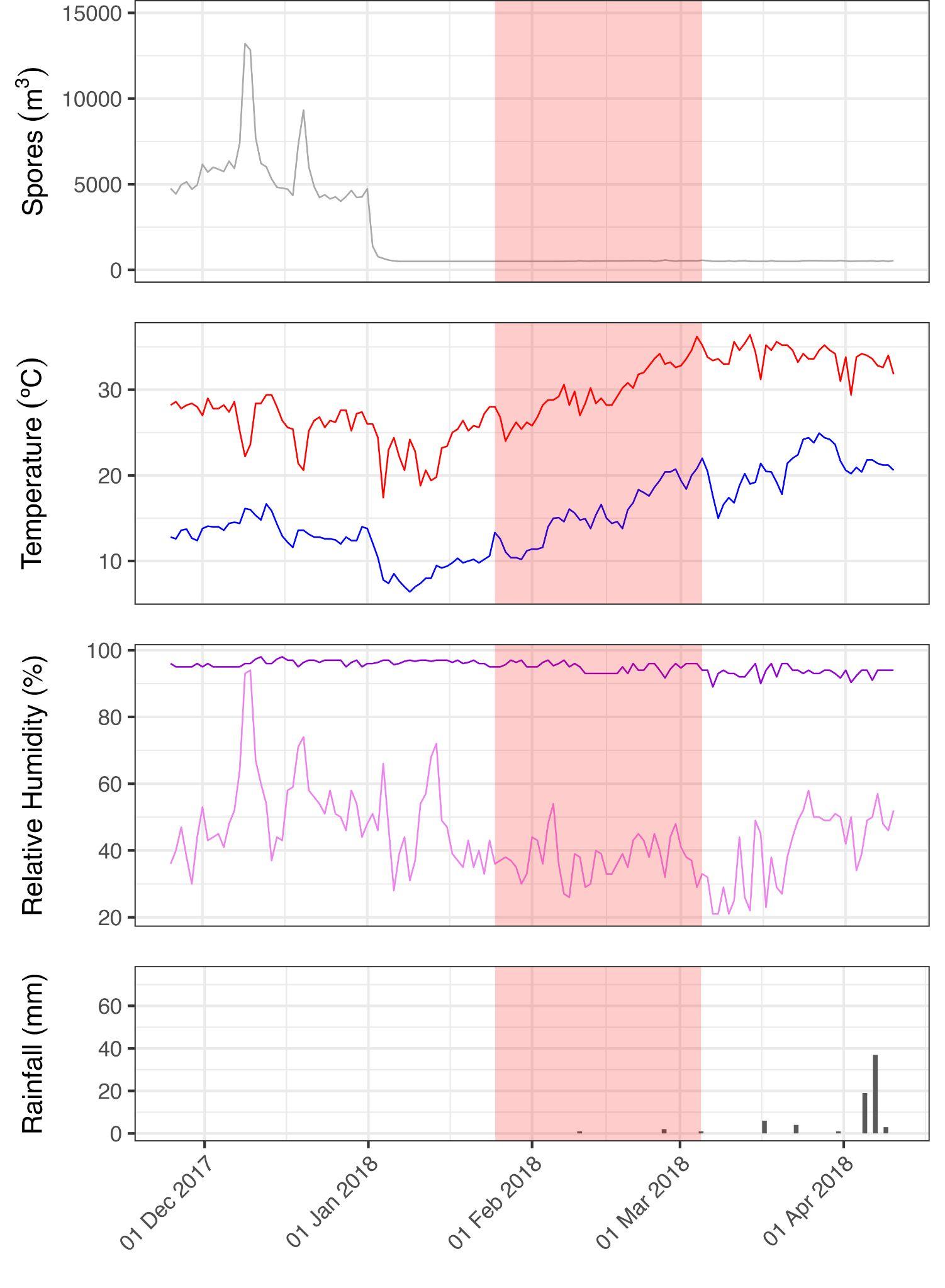 |
|  |
| 2018-19 Wheat Growing Season |
| 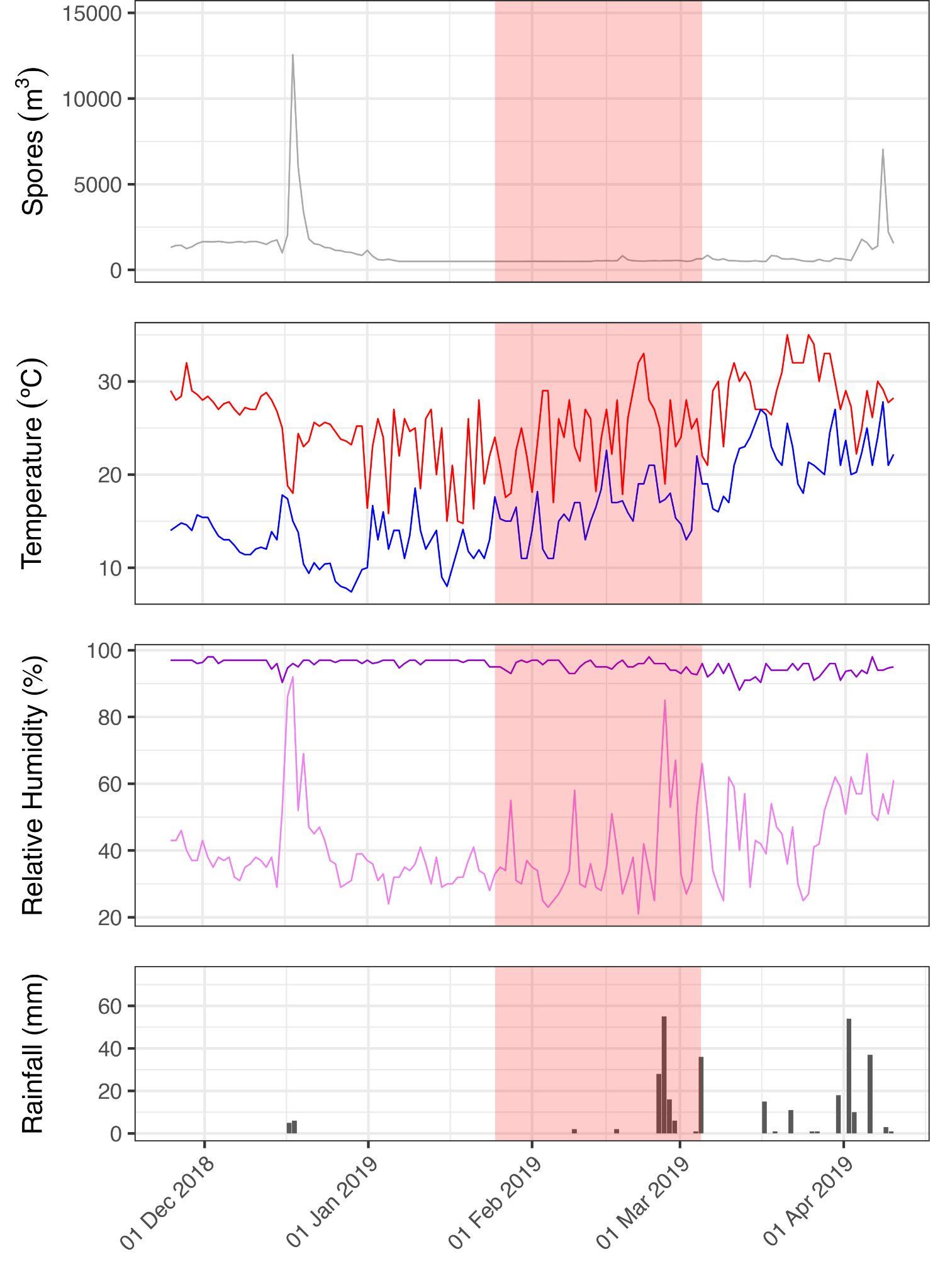 |

| 2019-20 Wheat Growing Season |
| --- |
| 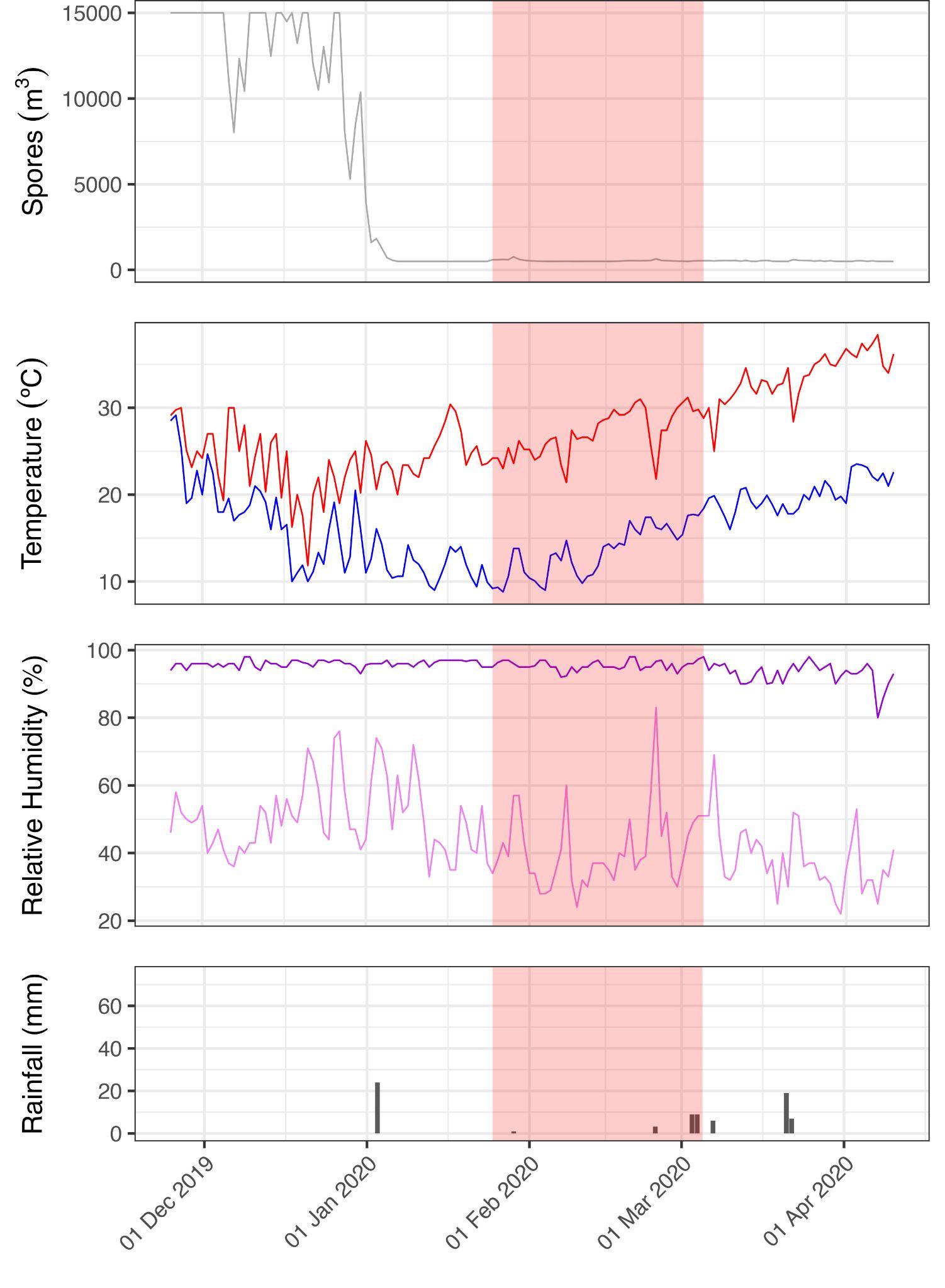 |

| 2020-21 Wheat Growing Season |
| --- |
| 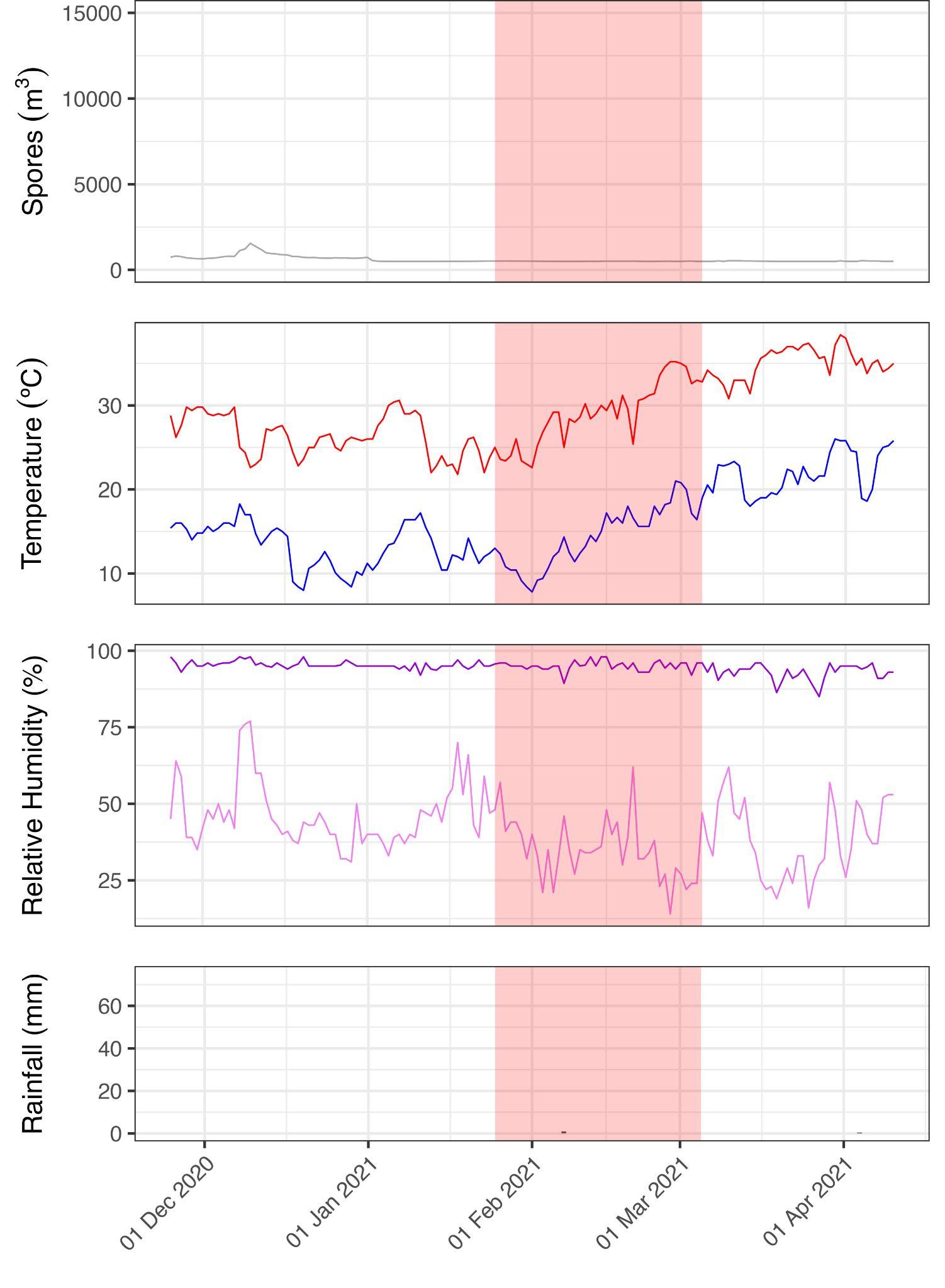 |

| 2021-22 Wheat Growing Season |
| --- |
| 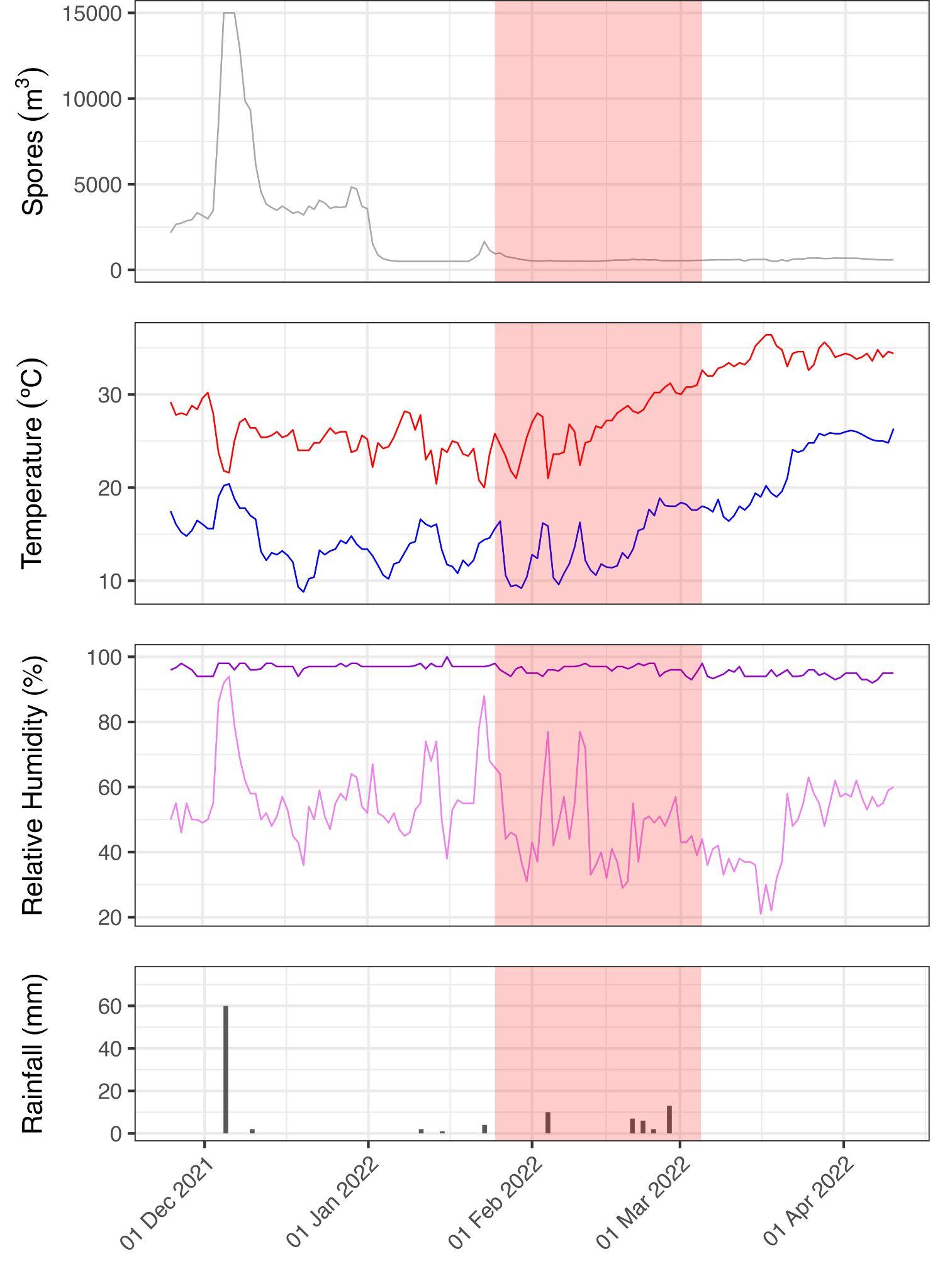 |

**Supplementary Materials 4.** The daily simulated conidia density (gray line) with accompanying weather data from the wheat growing seasons of 2016 through 2022 in Uberaba, Brazil. Note that 2017 and 2019 were epidemic years. The shaded area corresponds approximately to the heading stage window. Daily maximum temperature (red line) and minimum temperature (blue line), daily maximum relative humidity (dark violet line), daily minimum relative humidity (violet line), and daily accumulated rainfall (vertical bars)**.**

| 2016 Wheat Growing Season |
| --- |
| 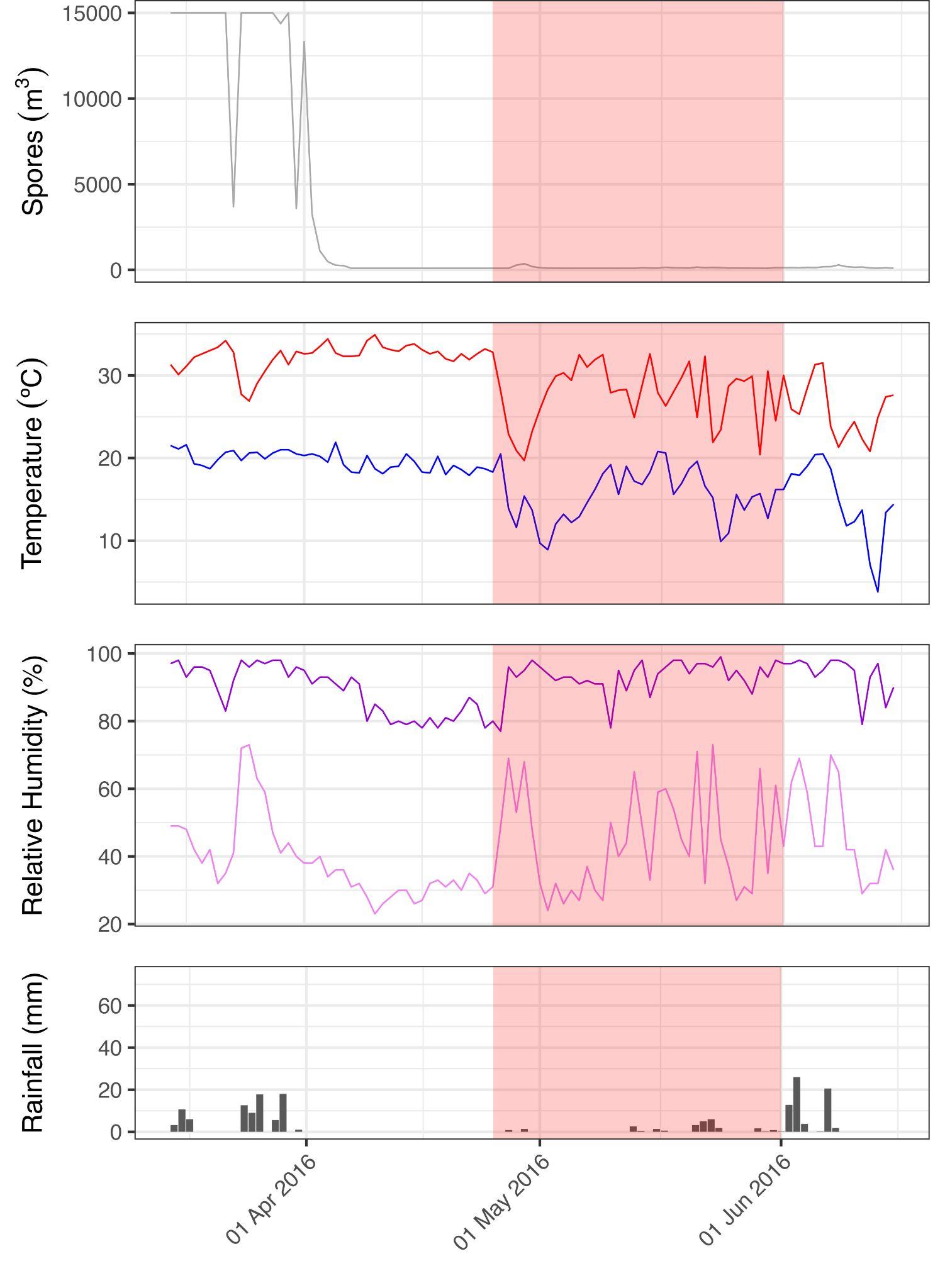 |
| 2017 Wheat Growing Season |
| 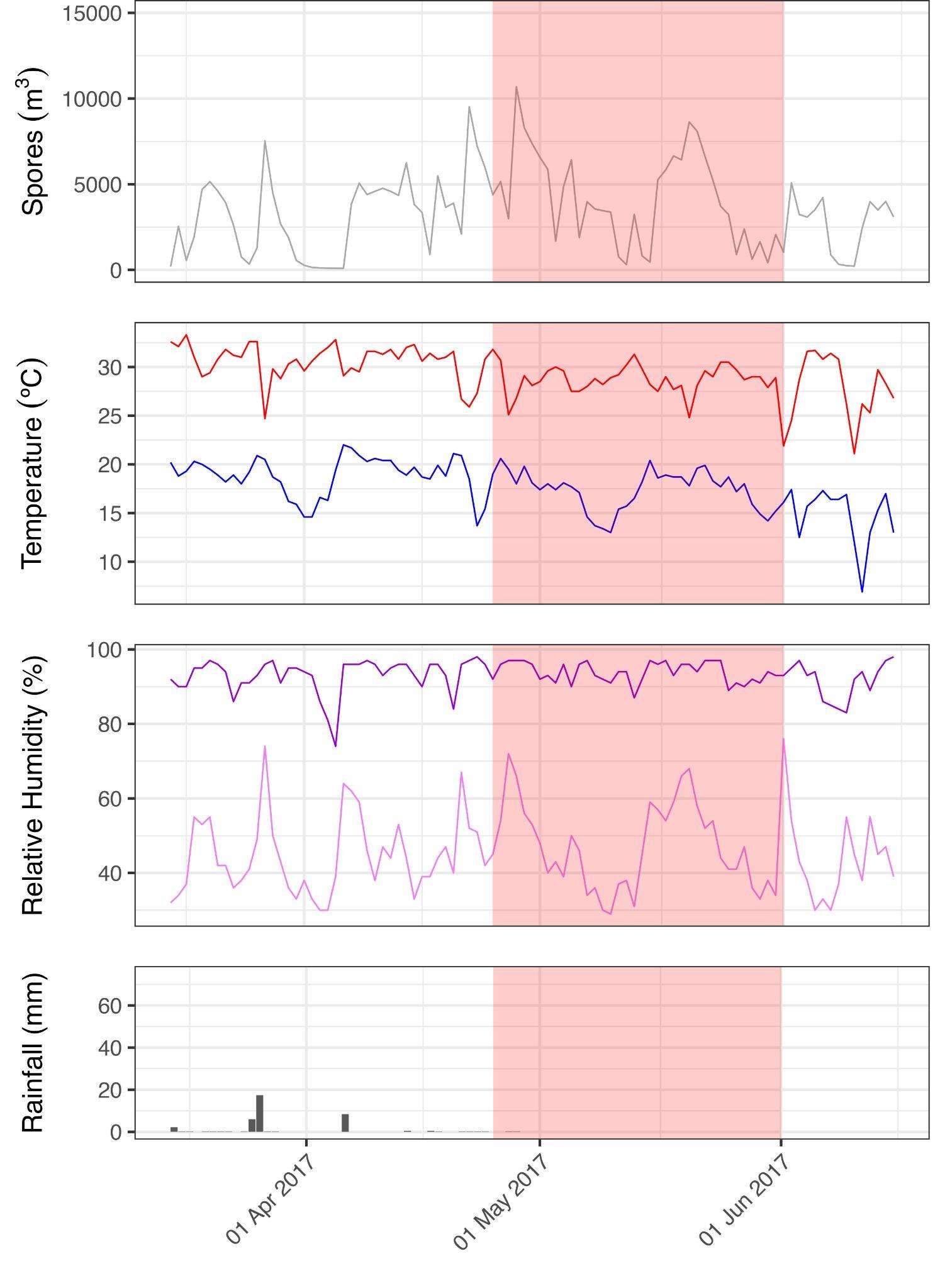 |
| 2018 Wheat Growing Season |
| 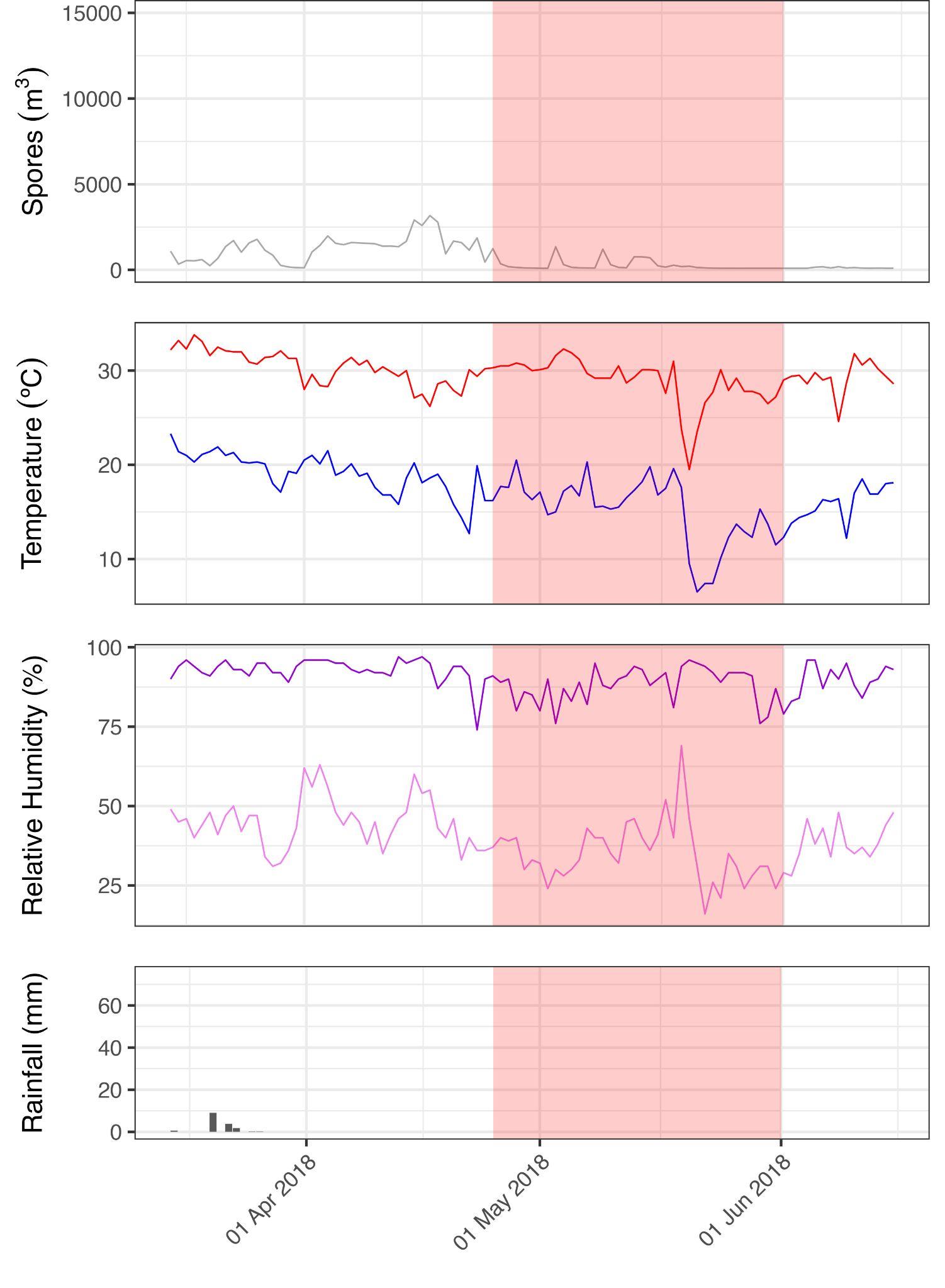 |

| 2019 Wheat Growing Season |
| --- |
| 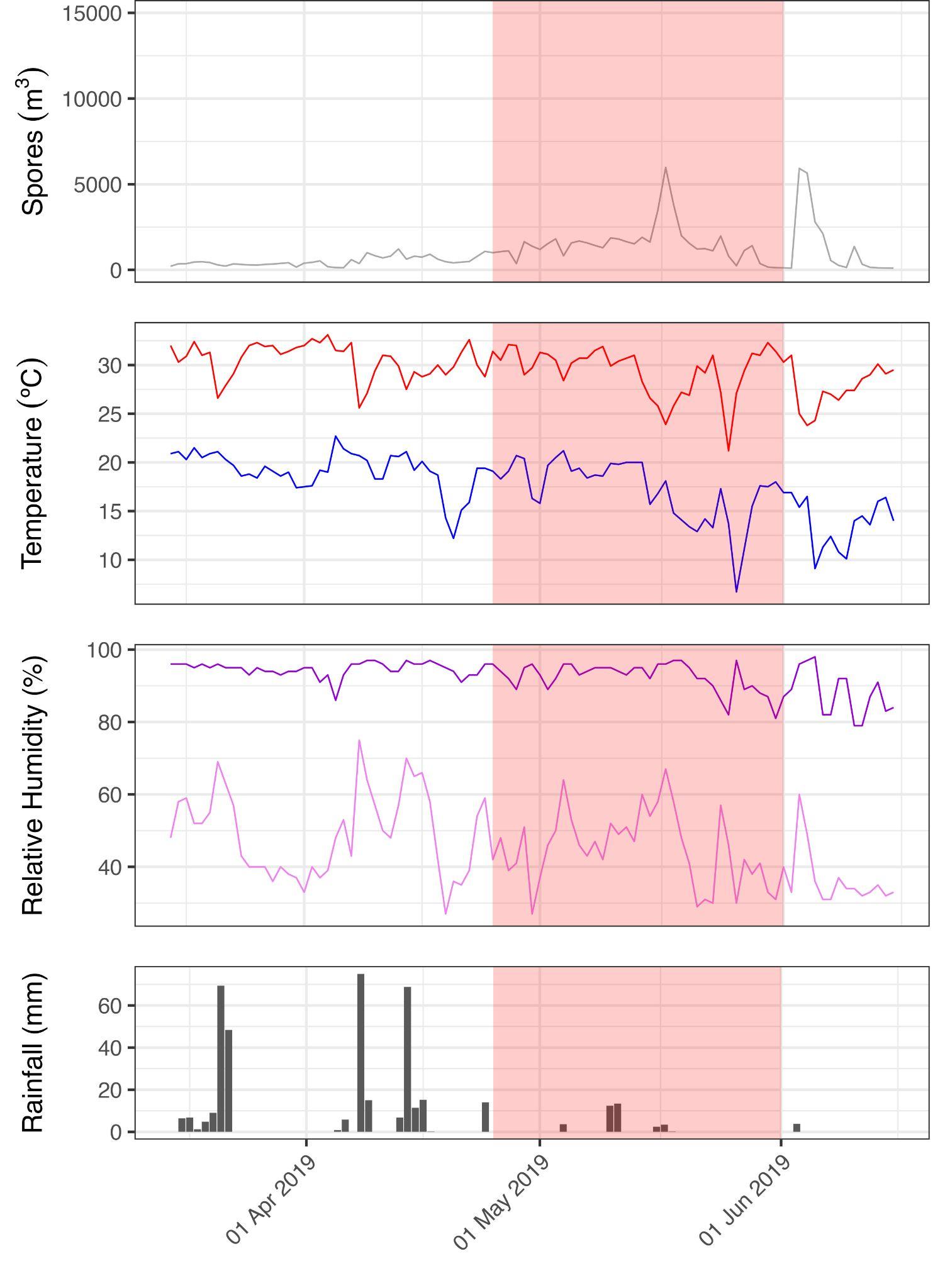 |

| 2020 Wheat Growing Season |
| --- |
| 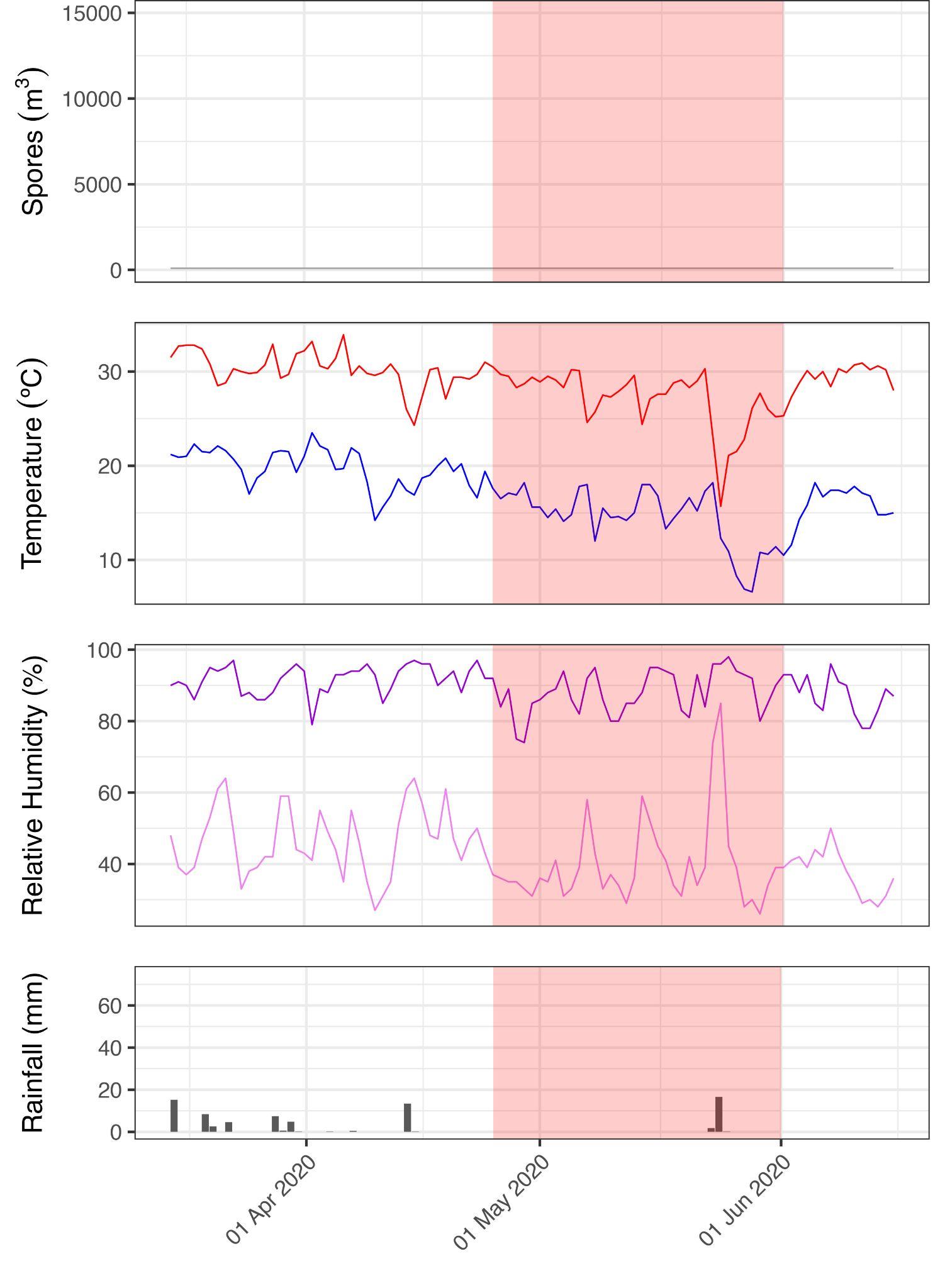 |

| 2021 Wheat Growing Season |
| --- |
| 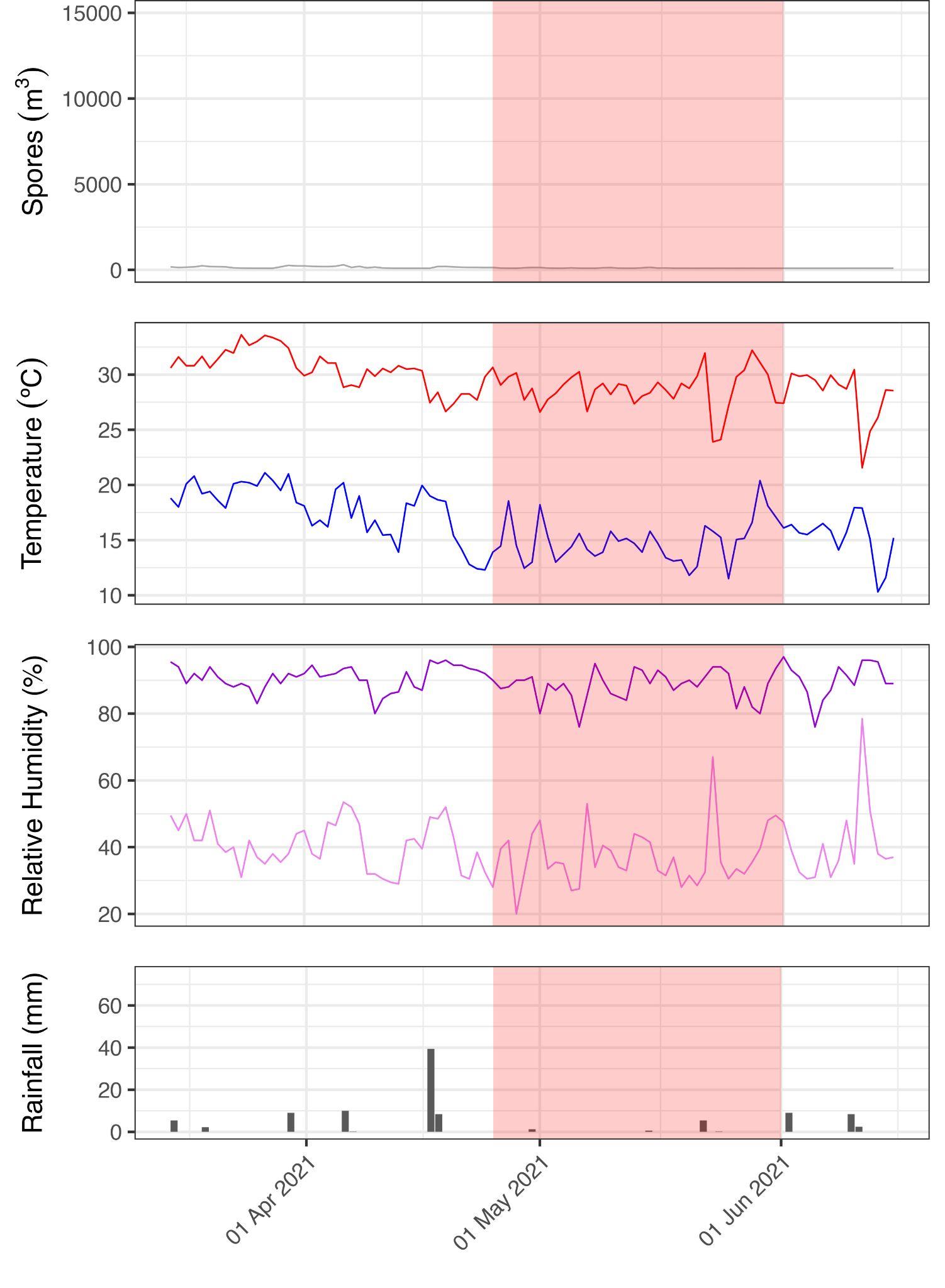 |
